# Selenoprotein N is an endoplasmic reticulum calcium sensor that links luminal calcium levels to a redox activity

**DOI:** 10.1073/pnas.2003847117

**Published:** 2020-08-17

**Authors:** Alexander Chernorudskiy, Ersilia Varone, Sara Francesca Colombo, Stefano Fumagalli, Alfredo Cagnotto, Angela Cattaneo, Mickael Briens, Mireille Baltzinger, Lauriane Kuhn, Angela Bachi, Andrea Berardi, Mario Salmona, Giovanna Musco, Nica Borgese, Alain Lescure, Ester Zito

**Affiliations:** ^a^Istituto di Ricerche Farmacologiche Mario Negri IRCCS, 20156 Milan, Italy;; ^b^Institute of Neuroscience, Consiglio Nazionale delle Ricerche and BIOMETRA Department, Università degli Studi di Milano, 20129 Milan, Italy;; ^c^Proteomics/MS Facility, Cogentech SRL Benefit Corporation, 20139 Milan, Italy;; ^d^Architecture et Réactivité de l’ARN, CNRS, Université de Strasbourg, 67084 Strasbourg, France;; ^e^IFOM-FIRC Institute of Molecular Oncology, 20139 Milan, Italy;; ^f^Biomolecular NMR Unit, IRCCS Ospedale S. Raffaele, 20132 Milan, Italy

**Keywords:** calcium sensor, SEPN1, endoplasmic reticulum, stress of the endoplasmic reticulum

## Abstract

The endoplasmic reticulum (ER) is the reservoir for calcium in cells. Luminal calcium levels are determined by calcium-sensing proteins that trigger calcium dynamics in response to calcium fluctuations. Here we report that Selenoprotein N (SEPN1) is a type II transmembrane protein that senses ER calcium fluctuations by binding this ion through a luminal EF-hand domain. In vitro and in vivo experiments show that via this domain, SEPN1 responds to diminished luminal calcium levels, dynamically changing its oligomeric state and enhancing its redox-dependent interaction with cellular partners, including the ER calcium pump sarcoplasmic/endoplasmic reticulum calcium ATPase (SERCA). Importantly, single amino acid substitutions in the EF-hand domain of SEPN1 identified as clinical variations are shown to impair its calcium-binding and calcium-dependent structural changes, suggesting a key role of the EF-hand domain in SEPN1 function. In conclusion, SEPN1 is a ER calcium sensor that responds to luminal calcium depletion, changing its oligomeric state and acting as a reductase to refill ER calcium stores.

## Significance

Selenoprotein N (SEPN1) is a type II transmembrane protein of the endoplasmic reticulum (ER) that senses luminal calcium through an EF-hand domain. On calcium depletion, a SEPN1 oligomer, prevalent under basal calcium concentrations, dissociates to generate a monomeric polypeptide that has enhanced redox trapping potential for its target the calcium pump, SERCA2, as well as for many additional interactors, indicating enhanced reductase activity. Our studies not only support that SEPN1 is one of the long-sought reductases of the ER, but also identify a feedback mechanism through which SEPN1 senses the luminal calcium level to modulate downstream signal transduction. Our results suggest that SEPN1 regulates the SERCA-mediated replenishment of ER calcium stores, a crucial mechanism for excitation-contraction coupling in skeletal muscle.

Calcium is an important second messenger that mediates a plethora of functions, ranging from muscle contraction to neurotransmitter release and egg fertilization (1). This ion has a steep concentration gradient across the plasma membrane and the different intracellular membranes, with a cytosolic concentration four orders of magnitude lower than that of the extracellular space or the lumen of the endoplasmic reticulum (ER). Of note, the release of calcium from the intraorganelle stores of the ER into the cytosol represents one of the most widely used signaling mechanisms of eukaryotic cells (2).

The ER and its specialized appendix in skeletal muscle, the sarcoplasmic reticulum (SR), are the cellular reservoir for calcium, the concentration of which ranges from 100 μM to 1 mM (3, 4). Such high concentrations compared with the low nanomolar concentration in the cytosol are maintained by the activity of sarcoplasmic/endoplasmic reticulum calcium ATPase (SERCA) pumps, which are ER membrane proteins that force calcium entry into the ER by hydrolyzing ATP. Three differentially expressed genes encode at least five isoforms of the SERCA pump: SERCA1a, SERCA1b SERCA2a, SERCA2b, and SERCA3. Among these isoforms, SERCA2b interacts with the oxidoreductases ERp57 and ERdj5, which modulate its activity of ER calcium reuptake (5, 6).

SERCA activity is opposed by inositol triphosphate receptor (IP3R) and ryanodine receptor (RYR), which instead determine calcium release from the ER (7, 8). The activity of both the SERCA pump and IP3R and RYR are regulated by redox, indicating a cross-talk between the redox state of calcium handling proteins and their activity in regulating luminal calcium levels (5, 9–12).

Calcium levels in the ER are tightly regulated to maintain an environment suitable for protein folding and to maintain the steep gradient across the ER membrane required for rapid excitation-contraction coupling. To defend appropriate calcium levels, the ER is equipped with a calcium-sensing mechanism. One of the main components of this mechanism is the ER membrane protein STIM1. Through the EF-hand domain, STIM1 senses the reduced calcium luminal level that occurs in skeletal muscle during excitation-contraction coupling and activates store-operated calcium entry (SOCE), increasing ER uptake of calcium from the extracellular space (13, 14). While this phenomenon is key for the ER/SR to retrieve calcium from the extracellular space, cells, and particularly muscle cells, must also be able to rapidly restore basal low cytosolic calcium concentrations to allow the contractile apparatus to rapidly return to the resting state after contraction (15). However, no calcium sensor in the ER/SR that could directly connect luminal calcium levels with the transfer of cytosolic calcium has been identified so far.

We previously reported that SEPN1 is a ubiquitously expressed protein (16, 17) that activates SERCA2-mediated calcium uptake into the ER in a redox-dependent manner, and that its loss of function gives rise to a congenital myopathy known as SEPN1-related myopathy (18). Here we extend our earlier findings showing that SEPN1, a type II transmembrane protein, is a calcium sensor of the ER. The EF-hand domain in SEPN1 is localized in the ER lumen and leads this protein to bind calcium with an affinity constant in the range of the ER calcium concentration. In vivo and in vitro experiments show that low luminal calcium triggers a conformational change in SEPN1, activating it as a reductase. Single amino acid mutations in the EF-hand domain of SEPN1, indexed in the clinical genomic variation database ClinVar, affect SEPN1 calcium affinity and its conformational change. Our findings identify a feedback mechanism through which SEPN1 senses luminal calcium levels and consequently regulates the ER redox poise, thereby modulating downstream signal transduction and the SERCA-mediated replenishment of ER calcium stores.

## Results

### SEPN1 Is an ER Type II Membrane Protein.

**Fig. 1.**
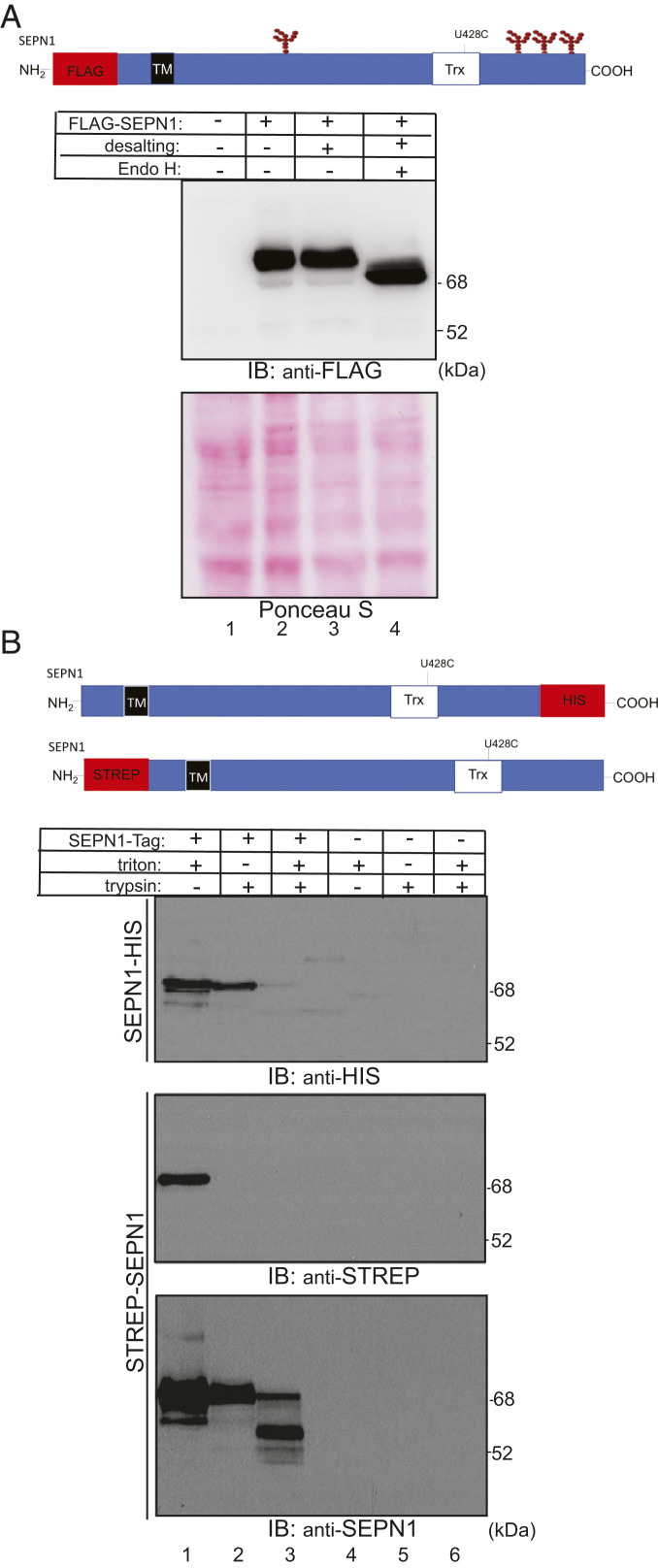
SEPN1 is a type II ER membrane protein. (*A*) Schematic representation of the recombinant SEPN1 construct exploited in the experiments related to this panel and indicating the four glycosylation sites: Asn156, Asn449, Asn471, and Asn497. FLAG Immunoblot representing FLAG-SEPN1 from transfected cells from which proteins were extracted with lysis buffer, the buffer was exchanged by PD-10 desalting column and the proteins digested by EndoH. Ponceau stain served as a protein loading control. (*B*) Schematic representation of the two recombinant SEPN1 constructs used in the experiments related to this panel. Human SEPN1 was fused either to a His-6-tag at its C-terminal (SEPN1-HIS) or to a Strep-tag at its N-terminal end (STREP-SEPN1). The TM domain is the predicted transmembrane domain between amino acids 31 and 50, as predicted by TMHMM Server version 2.0; the U428C mutation is indicated. HeLa cells expressing SEPN1-HIS or STREP-SEPN1 or untransfected were subjected to anti-STRP, anti-HIS, and anti-SEPN1 antibody detection. Membrane-enriched protein fractions were treated with detergent Triton-X100, followed by limited trypsin digestion. Microsome-enriched fractions were subjected to trypsin digestion in the absence of the detergent, to maintain membrane integrity. Both anti-tags and anti-SEPN1 detection indicated the expression of an intact SEPN1 of ∼75 kDa. Trypsin treatment applied to the native membrane fractions removed the N-terminal Strep-tag but preserved the His-tag. Both tags were digested in the detergent-dissociated membrane fractions treated with trypsin, and short-degraded forms of SEPN1 were detected.

Previous studies have identified SEPN1 as an integral membrane protein of the ER (17). To determine the type of membrane insertion, we first carried out a bioinformatics sequence analysis using software tools for predicting a transmembrane domain, including SOSUI, SPLIT, PSIPRED, and TMHMM. All of the algorithms used produced a consensus output, revealing the presence of a single transmembrane domain in the N-terminal part of the SEPN1 sequence and thus suggesting that SEPN1 is a type II transmembrane protein. To experimentally verify SEPN1 topology, we first checked its *N*-glycosylation status. Examination of SEPN1 amino acid sequences using dedicated bioinformatics tools predicted the presence of four putative glycosylation sites in SEPN1 at positions Asn156, Asn449, Asn471, and Asn497 (Fig. 1*A*). As the active site of the oligosaccharyl-transferase complex is located in the ER lumen, the use of any one of these sites would demonstrate SEPN1 translocation across the ER membrane.

To assess possible *N*-glycosylation of SEPN1, we analyzed FLAG-tagged SEPN1 by immunoblotting, after treatment of cell lysate with the enzyme endoglycosidase H (Endo H), which cleaves off *N*-linked oligosaccharides. As shown in Fig. 1*A* (lane 4), Endo H treatment caused a shift of the SEPN1 band to a faster migrating species, demonstrating the presence of *N*-glycosylation sites in the lumen. In agreement with a type II topology of SEPN1, mass spectrometry (MS) analysis of human SEPN1 heterologously expressed in *Pichia pastoris* demonstrated the presence of high-mannose glycosylation at Asn156, Asn449, and Asn497 (*SI Appendix*, Fig. S1 *A* and *B*).

To confirm the type II topology, a protease protection assay was carried out on HeLa cells transfected with SEPN1 carrying either a C-terminal HIS tag or an N-terminal STREP tag (Fig. 1*B*). Membrane fractions prepared from these cells were digested with trypsin in the presence or absence of detergent. Protein regions residing outside the ER are accessible to protease, whereas luminal protein domains remain protected but are degraded when the ER membrane is disrupted by detergent treatment. As shown in an immunoblot in Fig. 1*B* (*Middle*, lanes 1 and 2), the N-terminal STREP-tag was completely degraded by trypsin in the absence of detergent. Probing the same blot with an anti-SEPN1 antibody revealed that this treatment caused degradation of the tag but not of the majority of the polypeptide (Fig. 1*B*, *Lower*, lanes 1 and 2). In contrast, the C-terminal HIS tag was largely protected from trypsin attack in the absence of detergent (Fig. 1*B*, *Upper*). As expected, the protected band disappeared almost completely under conditions of simultaneous trypsin and detergent treatment (Fig. 1*B*, *Upper*, lane 3), and smaller fragments were revealed by the anti-SEPN1 antibody under this condition (Fig. 1*B*, *Lower*, lane 3). Notably, only a slight shift in SEPN1 migration was observed after digestion (Fig. 1*B*, *Upper*, compare lanes 1 and 2), as would be expected if the exposed cytosolic N-terminal domain were limited to the 30 amino acids preceding the predicted transmembrane domain (amino acids 31 to 50). These results confirm the type II topology of SEPN1 predicted by software analysis tools and by the experimental verification of its glycosylation.

### Calcium-Dependent Conformational Change of SEPN1.

Having established the topology of SEPN1, we used bioinformatics tools to search for known domains within the luminal sequence. This analysis predicted the presence of a sequence resembling the well-characterized calcium-binding domain, known as EF-hand, together with a thioredoxin (Trx) domain encompassing the selenocysteine residue.

Canonical EF-hands usually are composed of a helix-loop-helix structure with two well-defined alpha helices that allow calcium ion coordination in a pentagonal bipyramidal configuration (19). The lack of a solved 3D structure for SEPN1 makes it impossible to evaluate the architecture of its EF-hand directly; therefore, we opted for ab initio structure prediction using the QUARK algorithm (https://zhanglab.ccmb.med.umich.edu/QUARK/) (20). Modeling of the SEPN1 EF-hand structure reveals a single α-helix followed by flexible loop and β-sheet portion instead of a second α-helix. Therefore, despite the high sequence similarity to an EF-hand, the putative SEPN1 EF-hand may have an uncommon structure.

**Fig. 2.**
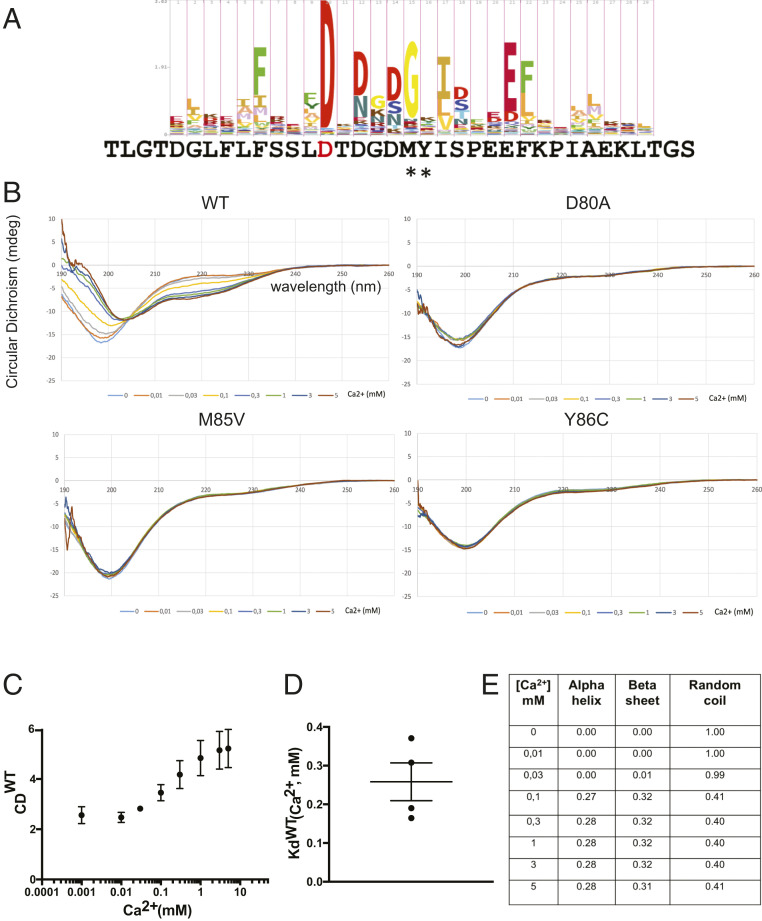
Ca^2+^ binding and Ca^2+^-dependent conformational change in SEPN1. (*A*) Alignment of SEPN1 predicted EF-hand sequence to the EF-hand HMM logo plot (obtained from PFAM; https://pfam.xfam.org/family/PF00036#tabview=tab4). The important conserved amino acid D80 within the SEPN1 sequence, depicted in red, was mutated to A. Pathogenic mutants identified within this sequence are highlighted by a star and correspond to the mutants M85V and Y86C. Residue numbering is based on UniProt entry Q9NZV5. (*B*) CD spectra of the indicated peptides at different calcium concentrations (0 to 5 mM). (*C*) CD (mdeg) values at 220 nm of the WT peptide at different calcium concentrations. (*D*) *K*_d_^Ca2+^ of WT calculated from CD values at 220 nm in four different experiments with different peptide preparations. (*E*) Analysis of the secondary structure of the WT by the K2D algorithm (dichroweb.cryst.bbk.ac.uk/).

The predicted difference between the simulated structure of the EF-hand in SEPN1 and the structures of canonical EF-hands raised the question of whether this domain is functional and able to bind calcium. To solve this conundrum, we generated synthetic peptides of 36 amino acids corresponding to a wild-type (WT) SEPN1 EF-hand and three mutants with single amino acid substitution: D80A, bearing a mutation of the most important calcium coordinating residue in canonical EF-hands, and two mutants, M85V and Y86C, described in the ClinVar database as genomic variations possibly associated with SEPN1-related myopathy (https://www.ncbi.nlm.nih.gov/clinvar/variation/195363/ and https://www.ncbi.nlm.nih.gov/clinvar/variation/461631/) (Fig. 2*A*). These peptides were subjected to calcium titration (0 to 5 mM), during which peptide conformational changes were monitored by measuring molecular ellipticity with circular dichroism (CD). The CD spectra for WT revealed significant calcium-dependent conformational changes. In contrast, CD spectra for D80A, M85V, and Y86C did not show any conformational change even at calcium concentrations as high as 5 mM (Fig. 2*B*).

The *K*_d_ value of WT for calcium, calculated from four independent experiments with different peptide preparations, was estimated as 242 ± 50 μM (mean ± SEM) (Fig. 2 *C* and *D*), a value in line with luminal calcium concentration (21). Similar *K*_d_ values of WT for calcium was obtained by measuring intrinsic tyrosine fluorescence of the peptide (193 ± 11 μM) (*SI Appendix*, Fig. S2 *A*–*D*) and by isothermal calorimetry (ITC) (129 ± 4 μM) (*SI Appendix*, Fig. S2 *E* and *F*). Isothermal calorimetry also established that the stoichiometry between SEPN1 and Ca^2+^ was 1:1 and confirmed the unresponsiveness of D80A to calcium (*SI Appendix*, Fig. S2 *E* and *F*). In addition, a deconvolution of secondary structure from the CD data using the DichroWeb K2D algorithm (dichroweb.cryst.bbk.ac.uk/) (22) confirmed that WT peptide was completely unstructured before calcium addition (100% random coil) but adopted a more structured form during calcium titration, including an increase in α-helix content up to 28% and up to 32% for β-sheets after addition of 300 μM calcium, which recalls the best predicted structures by QUARK (Fig. 2*E*).

Taken together, these data suggest that the WT peptide binds calcium with an affinity constant in the concentration range of ER calcium and undergoes marked conformational changes on calcium binding, different that those seen in the three mutants that do not respond to calcium (up to 5 mM of calcium).

### Calcium-Dependent Oligomerization of SEPN1.

**Fig. 3.**
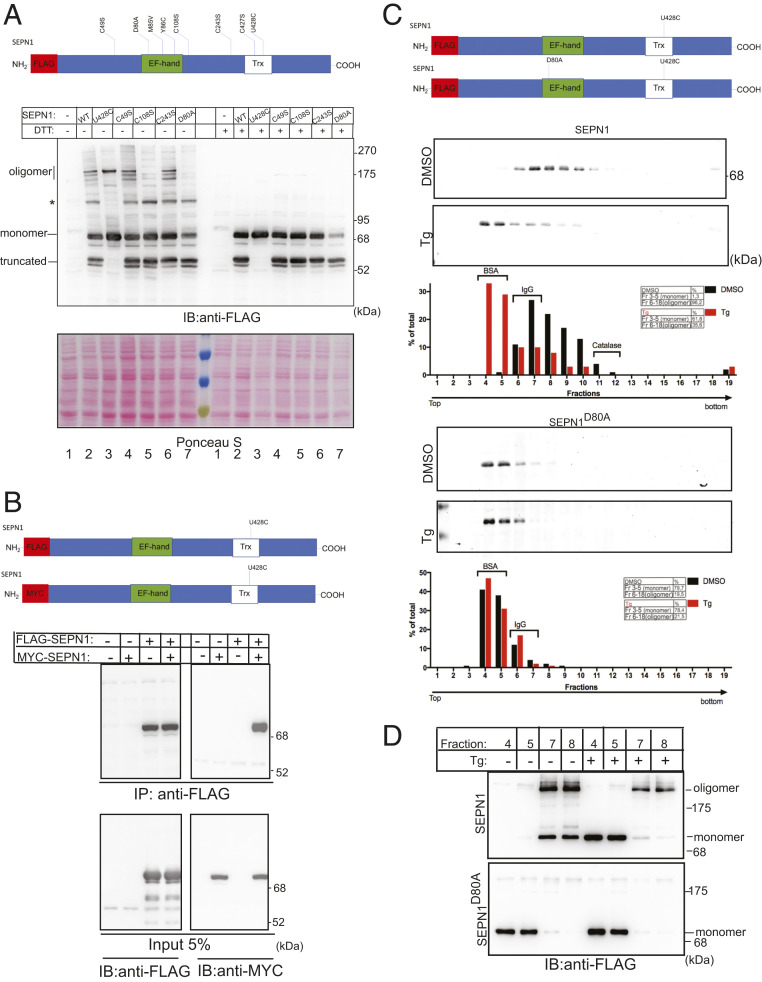
Ca^2+^-dependent oligomeric change of SEPN1 in cells. (*A*) Scheme of FLAG-SEPN1 protein and its mutants. The EF-hand domain, the Trx domain, and all the amino acid mutants are noted. Below are nonreducing and reducing FLAG immunoblots of the indicated FLAG-SEPN1 and its mutants. The truncated, monomeric, and oligomeric SEPN1 are indicated. The asterisk indicates an additional band that is visible for the selenocysteine-containing form and whose origin was not analyzed. Membrane staining with Ponceau S is shown as protein-loading control. (*B*) Scheme of FLAG-SEPN1 and MYC-SEPN1 protein. Immunoblot of MYC-tagged SEPN1 immunopurified with FLAG-M2 antibody from lysate of HeLa cells that were untransfected or transfected with expression plasmids of the indicated proteins. The lower two panels represent 5% of the total input protein lysate immunopurified. The proteins were resolved on reducing SDS/PAGE. (*C*) Sucrose gradient analysis of FLAG-SEPN1 and FLAG-SEPN1^D80A^ in HeLa cells treated for 2 h with DMSO or Tg. Samples were analyzed on 4% to 20% sucrose gradients. Equal aliquots of the 19 fractions were analyzed by FLAG immunoblotting. The positions and the molecular weight of size markers (BSA, IgG, and catalase) are established from Coomassie blue-stained gel. The tables on the right indicate the percentages of the monomer and the oligomer. (*D*) Nonreducing FLAG immunoblotting of the peak fractions of BSA (of similar size as the SEPN1 monomer) and IgG (of similar size as the oligomer) indicating that SEPN1 protomers are kept together to form the oligomer not only by a disulfide bridge (running as an oligomer), but also by weak interactions (running as a monomer).

To investigate the effect of calcium concentration on SEPN1 structure and activity in cells, we created a panel of FLAG-tagged SEPN1 mutants and expressed them in mammalian cells (Fig. 3*A*). By nonreducing immunoblot, SEPN1 appeared in three major bands, corresponding to a predominant form (apparent molecular weight [Mw] ∼76 kDa), compatible with a glycosylated monomer, a slowly migrating form (apparent Mw ∼195 kDa, labeled “oligomer”), as well as a shorter form (apparent Mw ∼54 kDa), presumably resulting from incomplete translation of SEPN1 mRNA. (The selenocysteine-encoding UGA codon can be also read as a stop codon by the translation machinery.) Indeed, this band was absent from the SEPN1^U428C^ mutant, in which the selenocysteine was replaced with the cysteine (Fig. 3*A*, lanes 2 and 3).

Also noted were multiple bands close to SEPN1 oligomers, which most likely arise from the association of SEPN1 with its truncated form, since the SEPN1^U428C^ mutant, without any truncated forms, displayed only one major SEPN1 oligomer band. Importantly, the capability of SEPN1 to form oligomers was abolished in the EF-hand mutant SEPN1^D80A^ (Fig. 3*A*, lane 7).

As shown in the right part of the blot in Fig. 3*A*, all of the higher molecular weight forms disappeared on DTT treatment, suggesting the involvement of disulfide bonds in the oligomerization. As the cysteine mutant of the Trx domain of SEPN1 (C427S; U/C428S) is still able to form SEPN1 oligomers, we hypothesized that the three cysteines residues upstream to this domain in the SEPN1 sequence (C49, C108, and C243) might be involved in disulfide bridge formation. To test the involvement of these cysteines in SEPN1 oligomerization, we mutagenized each of these residues with the redox inert amino acid serine and analyzed the mutants under reducing and nonreducing conditions (Fig. 3*A*, lanes 4 to 6). Only SEPN1^C108S^ lost the ability to oligomerize, indicating the involvement of this cysteine in disulfide bond formation (Fig. 3*A*, lane 5).

Because of the problem of the generation of the truncated form of SEPN1 carrying the selenocysteine UGA codon, all subsequent experiments were carried out with the SEPN1^U428C^ mutant; when lacking other mutations, we refer to this form simply as SEPN1 and specify only additional engineered mutations.

To investigate whether SEPN1 self-association underlies the generation of high molecular weight species (oligomers), two differently tagged SEPN1 forms (MYC and FLAG) were coexpressed, and the ability of the anti-FLAG antibody to pull down the MYC-tagged SEPN1 was tested. The results of this coimmunoprecipitation experiment demonstrate the capacity of SEPN1 to self-associate, suggesting that the high molecular weight band contains two copies of the SEPN1 monomers (Fig. 3*B*). However, given its apparent molecular weight (∼195 kDa), this band could either represent an SEPN1 homodimer with anomalous sodium dodecyl sulfate polyacrylamide gel electrophoresis (SDS/PAGE) migration or contain additional components. Keeping this uncertainty in mind, we refer to it as an oligomer.

The absence of the oligomer in the D80A mutant (Fig. 3*A*, lane 7) suggested that the oligomeric state of SEPN1 might be regulated by calcium. To test this hypothesis, calcium was depleted from the ER compartment by a short (2-h) exposure of cells transfected with FLAG-tagged SEPN1 or with MYC-tagged SEPN1 to the irreversible SERCA inhibitor thapsigargin (Tg) or to the reversible SERCA inhibitor cyclopiazonic acid. Incubation with both inhibitors led to a shift of the monomer/oligomer ratio of SEPN1 in favor of the monomer (*SI Appendix*, Fig. S3 *A* and *B*).

To analyze the effect of calcium on the oligomerization of SEPN1 by an alternative method, we compared the sucrose gradient sedimentation profiles of FLAG-tagged SEPN1 and FLAG-tagged SEPN1^D80A^ (EF-hand mutant) in lysates from cells treated with Tg or untreated. In qualitative agreement with the results of the nonreducing immunoblot after Tg treatment (*SI Appendix*, Fig. S3*C*), FLAG-tagged SEPN1 was recovered mainly as a protein, with a molecular weight corresponding to a SEPN1 monomer (61.8% of the total) under Tg conditions, in a region overlapping the bovine serum albumin (BSA) (Mw 66.5 kDa) marker. In the absence of Tg treatment (DMSO-treated cells), SEPN1 instead was recovered nearly completely in its oligomeric form (96.2% of the total), sedimenting slightly ahead of the IgG marker (Mw 150 kDa) (Fig. 3*C*). Importantly, and in accordance with the results of nonreducing immunoblotting, the aggregation pattern of FLAG-tagged SEPN1 ^D80A^-transfected cells did not change under the two conditions of Tg (78.4% of the total monomer and 21.5% oligomer) and DMSO (79.7% of the total monomer and 21.5% oligomer). SEPN1 ^D80A^ was present almost exclusively as a monomer in both conditions, confirming that calcium binding to SEPN1 is a prerequisite for its monomer-to-oligomer conformational change (Fig. 3*C*).

The sucrose gradient analysis showed that a much higher proportion of SEPN1 is in an oligomeric state under basal ER luminal calcium concentrations than was revealed by the nonreducing gel analysis. This suggests that oligomer formation may be mediated by noncovalent bonds, and that only a portion of the oligomer is further stabilized by disulfide bonding. We confirmed this hypothesis by analyzing the peak monomer (fractions 4 and 5) and oligomer (fractions 7 and 8) fractions from the sucrose gradients by nonreducing SDS/PAGE immunoblotting. While SEPN1 after Tg treatment and its EF-hand mutant (D80A) treated or not treated with Tg showed only a monomer band, the oligomer peak fractions showed both monomer and oligomer bands, indicating dissociation of some of the oligomer under denaturing conditions (Fig. 3*D*).

Thus, both the sucrose gradient and the immunoblot analyses indicate that luminal calcium levels and calcium binding by the EF-hand regulate the oligomeric state of SEPN1.

### The Attacking Amino Acid of SEPN1 Is in a Reduced Form.

We previously showed that the mutant of SEPN1, SEPN1^C427S,^ ^U428C^, was able to trap interactors in a redox-dependent manner (18). Therefore, we investigated whether the attacking amino acid cysteine 428 (C428) is in a reduced form compatible with the redox state of an attacking amino acid in both monomeric and oligomeric forms of SEPN1 and in conditions of calcium depletion.

**Fig. 4.**
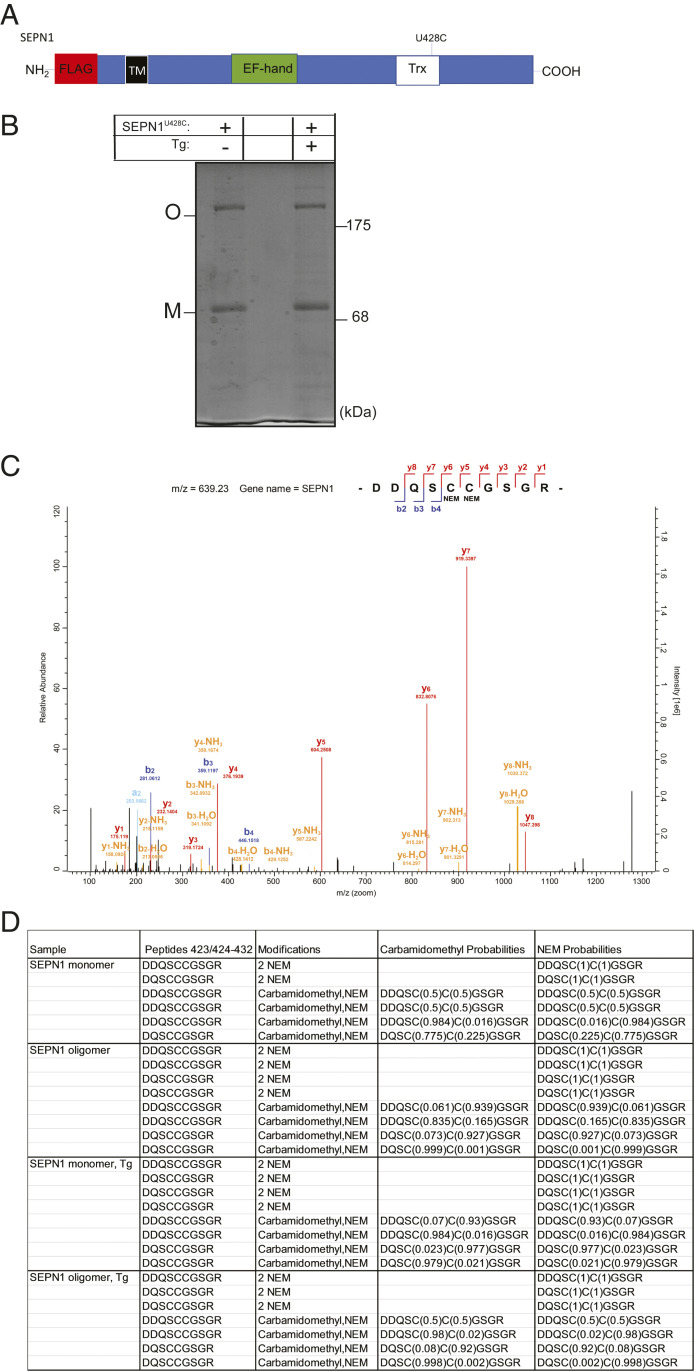
The attacking amino acid of SEPN1 in Trx domain is present in a reduced form. (*A*) Schematic representation of a SEPN1 construct exploited in the experiments related to this figure. (*B*) Coomassie blue-stained nonreducing SDS/PAGE of FLAG-immunopurified SEPN1 after treatment with Tg or DMSO indicating the bands of the monomeric (M) and oligomeric (O) form that were cut and the redox state of cysteines analyzed by nLC-ESI-MS/MS sequence analysis. Lane 1, transfected SEPN1^U428C^ and treated with DMSO; lane 2, empty; lane 3, transfected with SEPN1^U428C^ and treated with Tg. (*C*) Representative MS/MS spectrum for the peptide (423 to 432) derived from the monomer of SEPN1 and bearing NEM-alkylation of cysteines 427 and 428. (*D*) The redox state of cysteines 427 and 428 in SEPN1 after treatment with Tg or DMSO. Alkylation is reported as +NEM if the cysteines are alkylated by NEM and present in a reduced form or as +carbamidomethyl if the cysteines are alkylated by IAA and present in an oxidized form. The probability of different alkylation types (derived from MaxQuant analysis) is reported in parentheses.

HeLa cells transfected with FLAG-SEPN1^U428C^ and exposed to Tg or its vehicle DMSO were harvested, and the free thiols were N‐ethylmaleimide (NEM) alkylated (Fig. 4*A*). The protein lysates were then immunoprecipitated with FLAG M2 antibody and run on nonreducing SDS/PAGE. As expected, Coomassie staining of the gel revealed two main bands of FLAG-SEPN1 ^U428C^ corresponding to the monomer and oligomer. These two bands were excised from the gels, DTT-reduced, and then alkylated with iodoacetamide (IAA), which led to the labeling of the cysteines involved in disulfide bonds (Fig. 4*B*). The samples were then subjected to nano-scale liquid chromatography electrospray ionization tandem mass spectrometry (nLC-ESI-MS/MS) sequence analysis. The mass spectra of the fragmented peptides showed that, irrespective of Tg treatment, both the monomeric and the oligomeric species of SEPN1 contain the C428 alkylated by NEM, demonstrating that they are present in a reduced form compatible with that of a redox-attacking amino acid (Fig. 4 *C* and *D*).

### A Redox-Active SEPN1-Trapping Mutant Displays More Interactors upon Luminal Calcium Depletion.

**Fig. 5.**
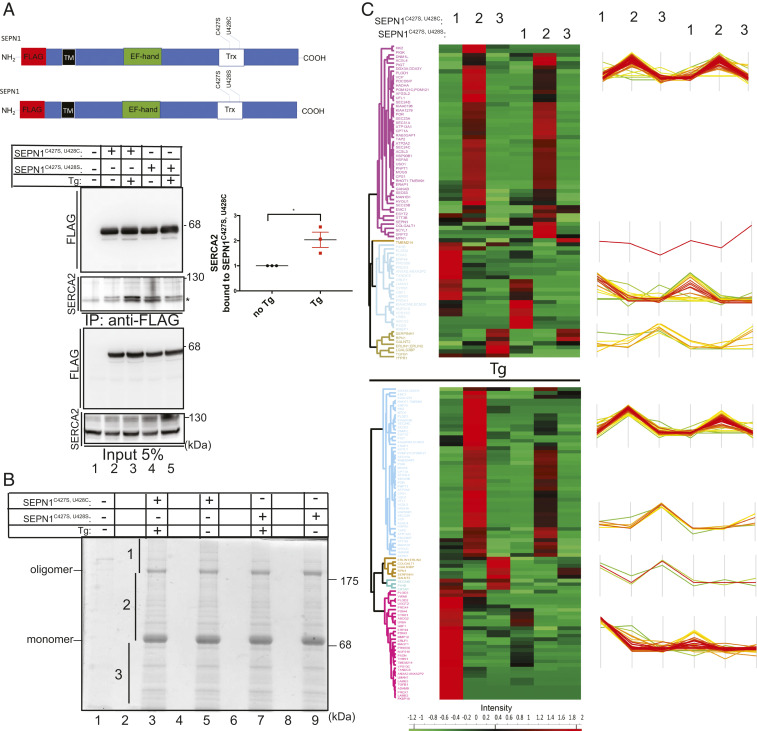
The SEPN1 trapping mutant in conditions of low luminal calcium engages a higher number of interactors. (*A*) Schematic representation of SEPN1 constructs exploited in the experiments of this figure. FLAG and SERCA2 immunoblots of FLAG-tagged SEPN1 immunopurified with FLAG-M2 antibody from lysate of cells that were untransfected or transfected with expression plasmids of the indicated proteins and treated with Tg or DMSO. The lower two panels represent the 5% of the total input protein lysate immunopurified. The proteins were resolved on reducing SDS/PAGE. The graph on the right shows the relative levels of SERCA2 associated with its bait, FLAG-SEPN1 (set to 1 in no Tg) in arbitrary units in three different experiments (*n* = 3; *P* < 0.05, unpaired *t* test). (*B*) Coomassie blue-stained nonreducing SDS/PAGE of FLAG-immunopurified SEPN1 from cells after treatment with Tg or DMSO indicating the bands of the monomeric and oligomeric form. Each lane was divided into three slices (1 to 3) that were cut and analyzed for protein identification by mass spectrometry. Lane 1, transfected with empty expression vector; lane 2, empty; lane 3, transfected with SEPN1^C427S,^ ^U428C^ and treated with Tg; lane 4, empty; lane 5, transfected with SEPN1^C427S,^ ^U428C^ and treated with DMSO; lane 6, empty; lane 7, transfected with SEPN1^C427S,^ ^U428S^ and treated with Tg; lane 8, empty; lane 9, transfected with SEPN1^C427S,^ ^U428S^ and treated with DMSO. (*C*) Unsupervised hierarchical clustering heat maps comparing SEPN1^C427S,^ ^U428C^ with SEPN1^C427S,^ ^U428S^ and SEPN1^C427S,^ ^U428C^ with SEPN1^C427S,^ ^U428S^ treated with Tg. Numbers 1 to 3 indicate the interactors identified in the slices 1 to 3 of the Coomassie blue-stained nonreducing SDS/PAGE in *B*.

To test whether in conditions of low luminal calcium, SEPN1 has a stronger trapping potential, we exploited the two mutants, FLAG-SEPN1^C427S,U428C^ and FLAG-SEPN1^C427S,U428S^. Previously, these two mutants led to the identification of SERCA2 (ATP2A2) as a redox-dependent interactor of SEPN1, and the interaction with SERCA2a and 2b isoforms was confirmed by coimmunoprecipitation experiments (18). The first of these mutants remains covalently bound to its substrate via Cys-428, while the second one, in which the attacking cysteine is mutated to a serine residue, serves as a control. We first asked whether the interaction between SEPN1 and SERCA2 was improved under conditions of luminal calcium depletion. HeLa cells were transfected with FLAG-SEPN1^C427S,U428C^ and FLAG-SEPN1^C427S,U428S^ and exposed to Tg or its vehicle DMSO. After the treatments, cells were harvested, and lysate was subjected to immunoprecipitation with FLAG M2 antibody. Immunoblot analysis showed that coimmunoprecipitation between FLAG-SEPN1^C427S,^ ^U428C^ and SERCA2 was enhanced under conditions of calcium depletion (Fig. 5*A*, lanes 2 and 3) and did not change between FLAG-SEPN1^C427S,^ ^U428S^ and SERCA2 (Fig. 5*A*, lanes 4 and 5) (18).

To test whether FLAG-SEPN1^C427S,^ ^U428C^ has a strong trapping potential in general under conditions of calcium depletion, protein lysates were immunoprecipitated with Flag M2 antibody, run on nonreducing SDS/PAGE, and stained with Coomassie blue. For both FLAG-SEPN1^C427S,^ ^U428C^ and FLAG-SEPN1^C427S,^ ^U428S^, this staining revealed two main bands corresponding to monomer and oligomer of SEPN1, along with a series of other bands throughout the lanes. Each lane was cut into three slices (designated 1 to 3) that underwent in-gel tryptic digestion; the eluted peptides were subjected to nLC-ESI-MS/MS sequence analysis, leading to the identification of the peptides belonging to SEPN1 interactors (Fig. 5*B*). The interactors were clustered into two heat maps, obtained by comparing the label-free quantification (LFQ) intensity of each single protein normalized to the SEPN1 signal (Fig. 5*C*). The heatmaps obtained from FLAG-SEPN1^C427S,^ ^U428C^ and FLAG-SEPN1^C427S,^ ^U428S^ in DMSO-treated cells showed no important difference in terms of abundance of interactors; instead, the abundance of interactors was strikingly in favor of FLAG-SEPN1^C427S,^ ^U428C^ when the cells had been exposed to Tg. Thus, the redox capability of SEPN1 is more pronounced in conditions of luminal calcium depletion.

Furthermore, the pathway analysis of SEPN1 interactors in conditions of luminal calcium depletion indicated enrichment in functional ontological terms corresponding to proteins of the response to ER stress (*SI Appendix*, Fig. S4). This suggests an important role of SEPN1 during ER stress response, as has been hypothesized (23, 24).

### The Reductive Shift in the ER Redox Poise Triggered by Calcium Depletion Is Absent in SEPN1 Knockout Cells.

To investigate the role of endogenous SEPN1, we generated SEPN1 knockout (KO) HeLa cells, using the CRISPR/CAS9 technology. The resulting SEPN1 KO cells had undetectable SEPN1, as revealed by immunoblotting with a specific SEPN1 antibody (*SI Appendix*, Fig. S5*A*).

**Fig. 6.**
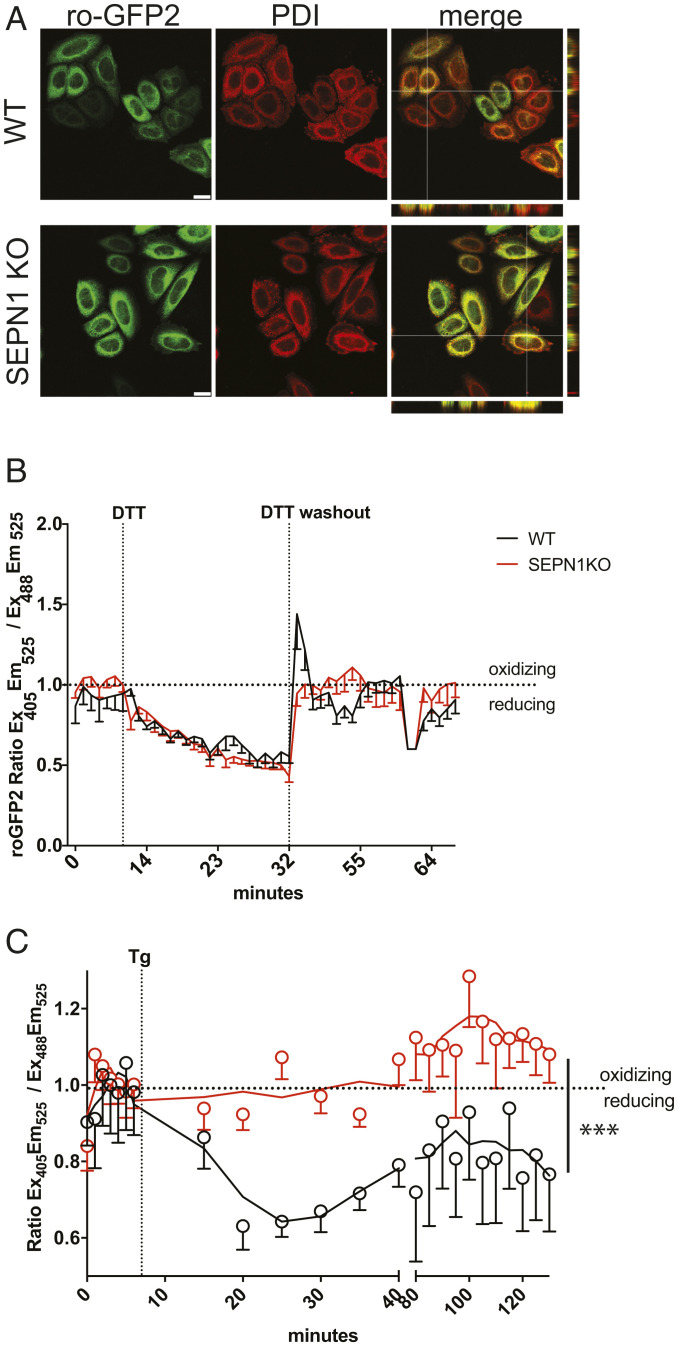
A reducing shift is lacking in SEPN1 KO cells after luminal calcium depletion. (*A*) Fluorescent photomicrographs of WT and SEPN1 KO cells transiently expressing an ER-localized roGFP2, immunostained for PDI as an ER marker. The merged panels with orthogonal views show an overlap of the roGFP2 signal with PDI (Scale bar: 20 μm.) (*B*) Traces of time-dependent changes in the fluorescence excitation ratio of roGFP2, reflecting the alterations in the redox state of roGFP2 localized in the ER of WT and SEPN1 KO cells. Cells were exposed to a DTT pulse of 20 min, followed by washout of the reductant. (*C*) Cells were exposed to the irreversible SERCA inhibitor Tg for 2 h. Each data point represents the mean ± SEM of the fluorescence excitation ratio of roGFP2. The experiment was reproduced five times, with similar results (*P* < 0.001, two-way ANOVA) (*SI Appendix*, Fig. S5*B*).

To track changes in ER redox poise, we took advantage of ER-localized roGFP2, a redox biosensor known to be a protein disulfide isomerase (PDI) client, and transfected it into WT and SEPN1 KO cells (25, 26). The roGFP2 colocalized with luminal ER PDI, confirming its ER localization in WT and SEPN1 KO cells (Fig. 6*A*). We next measured the redox changes of this sensor in live cells after exposure to DTT or Tg by comparing sensor emission intensity at 525 nm when excited at 405 nm (Ex_405_Em_525_) and 488 nm (Ex_488_Em_525_). The baseline redox signal of the ratio of Ex_405_Em_525_ to Ex_488_Em_525_ was set at 1; thus, a signal >1 or <1 after DTT or Tg indicated the oxidation or reduction of the sensor, respectively, compared with baseline.

As expected, ER roGFP2 was rapidly reduced after DTT treatment and oxidized to baseline values after washout of the reductant both in WT and SEPN1 KO cells. Moreover, WT and SEPN1 KO cells showed a similar redox profile of roGFP2 during the DTT treatment and its washout (Fig. 6*B*). A clear difference between WT and SEPN1 KO cells was instead observed in response to Tg treatment. As reported previously (26), in WT cells, the addition of Tg caused a progressive reduction of the redox sensor over a period of approximately 20 min, after which the probe remained in a reduced state. In contrast, the redox profile of roGFP2 in SEPN1 KO cells was not responsive to Tg (Fig. 6*C* and *SI Appendix*, Fig. S5*B*), suggesting that SEPN1 is an important player involved in the increased poise reduction of the ER lumen after calcium depletion and indicating that it plays a key role in adjusting the lumen’s redox poise to calcium levels.

## Discussion

SEPN1-related myopathy (SEPN1-RM) is a congenital disorder arising from loss-of-function mutations in the SEPN1 gene. It presents in infancy with heterogeneous clinical manifestations ranging from mild myopathy to severe muscle weakness that can lead to death due to respiratory failure (27).

Functional analyses using an SEPN1 KO mouse model suggested that the lack of SEPN1 leads to redox and calcium store impairment, thereby sensitizing skeletal muscle to oxidative insult and leading to chronic ER stress, which is part of the pathogenic mechanism of SEPN1-related myopathy (23, 24, 28, 29). However, the impact on the muscle phenotype of single SEPN1 mutations, which span the entire gene sequence in humans, has not yet been investigated and could possibly explain the clinical heterogeneity in SEPN1-RM.

A major obstacle to this lack of genotype-phenotype correlation is the paucity of data on SEPN1 function, owing in part to the difficulty in obtaining a pure and functionally active SEPN1, as it is a membrane and a selenocysteine-containing protein. As an alternative to the full-length protein, we used peptides containing the putative EF-hand domain of SEPN1 to probe for calcium-induced conformational changes and expressed the selenocysteine-to-cysteine variant of SEPN1 in cells to test whether the protein is activated as a reductase after ER calcium depletion. In this study, we were able to show that SEPN1 senses luminal calcium, and that when the level of this ion is above the range 100–300 μM, a conformational change in a more organized structure is induced in this protein. Furthermore, on luminal calcium depletion, a SEPN1 oligomer, which is prevalent under basal calcium concentrations, dissociates to generate the monomeric polypeptide. The self-association between SEPN1 protomers is mediated by noncovalent interactions and also by a disulfide bridge involving C108 immediately downstream from the EF-hand. Interestingly, this cysteine is not evolutionary conserved, as it appears only in the *Homo* lineage and is reported as a single nucleotide polymorphism in the respective sequence databases, with a low allele frequency in all populations (0.16; 0.30 in the highest population) according to 1000 Genomes data (http://www.ensembl.org/Homo_sapiens/Variation/Explore?db=core;r=1:25804663-25805663;v=rs7349185;vdb=variation;vf=3196487). This low frequency may suggest a potential detrimental effect of this cysteine variant, perhaps related to a dampened oligomer-monomer transition on calcium depletion.

In addition, we show that in conditions of low calcium, SEPN1 is present more as a monomer and presumably with the Trx domain more accessible to targets. Accordingly, we observed enhanced redox trapping potential of SEPN1, not only versus its target SERCA2, but also in general versus many other interactors, indicating enhanced activity as a reductase and a contribution to the ER stress response pathway, as suggested by the interaction with proteins belonging to this pathway. Thus, our studies support that SEPN1 is one of the long-sought reductases of the ER, and that its redox activity is regulated by calcium levels (30).

**Fig. 7.**
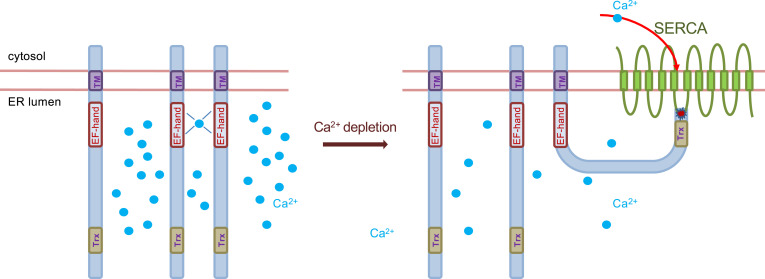
SEPN1 working model. Within the ER/SR, SEPN1 senses calcium levels by binding this ion through an EF-hand domain. When luminal calcium concentration drops (below 100–300 μM), the SEPN1 conformation changes and causes a shift toward a monomeric form, which is redox-active towards its partner (among others) SERCA. Activation of SERCA then leads to calcium entry into the ER and refilling of calcium stores.

We previously characterized SERCA2 isoforms as SEPN1 interactors in human cultured cells and showed a longer relaxation time after electrical stimulation in SEPN1-depleted flexor digitorum brevis muscle fibers, indicating reduced calcium entry in the SR, which is in good agreement with reduced SERCA activity in SEPN1 KO muscle. Thus, we can hypothesize an effect of SEPN1 on muscle and nonmuscle SERCA isoforms (23). Therefore, considering that SERCA2 is activated to pump calcium into the ER after reduction of the two luminal cysteines of its L4 domain, our results suggest that SEPN1 acts as an intermediary between ER calcium handling and redox regulation to refill the ER/SR calcium store in skeletal muscle (5, 6, 18, 23) (Fig. 7).

Our findings are particularly relevant to the pathogenic mechanism of SEPN1-RM, as all three single amino acid mutants of the EF-hand domain of SEPN1 that we analyzed displayed impaired calcium affinity and calcium-dependent conformational change, potentially affecting SEPN1 redox function. Similar to the SEPN1 mutants investigated here, mutations in the EF-hand of STIM1 affect calcium binding and lead a pathogenic phenotype due to constitutive STIM activation (31). Further analysis will be important to determine whether the mutations in the EF-hand of SEPN1 similarly lead to constitutive reductase activation.

## Materials and Methods

### Cell Culture and Transfection.

SEPN1 KO HeLa cells were generated using CRISPR/Cas9 technology (Origene) following the manufacturer’s guidelines. pCas-guide constructs encoding Cas9 and a custom guide RNA sequence (GAA​CTG​GCG​CTG​AAG​ACC​CT) targeting exon 2 of the human SEPN1 gene were ordered from Origene. 293TN and HeLa cells were cultured in DMEM (Gibco) supplemented with 2 mM glutamine, 10% fetal bovine serum, and 1% penicillin-streptomycin in a humidified atmosphere of 5% CO_2_ at 37 °C.

The cells were transfected at 20% to 50% confluence with FuGENE HD transfection reagent (Promega) under optimized conditions. In brief, 3 μL of reagent was used for every 1 μg of DNA, and the transfection mix was prepared in Opti-MEM (Gibco).

For experiments with His- and Strep-tagged SEPN1, cells were grown in six-well plates and transiently transfected at 80% confluency with nanofectin (GE Healthcare) in accordance with the manufacturer’s recommendations. Cell-based assays were performed in biological triplicate.

### SEPN1 Protease Protection Assay.

At 48 h posttransfection, HeLa cells were washed with PBS and scraped in 100 μL of PBS, followed by centrifugation at 600 × *g* for 5 min at 4 °C to pellet cells. The cells were lysed in 100 μL of buffer A (0.25 M sucrose and 5 mM Hepes pH 7.4). Protein concentration was then determined using a standard Bradford protocol.

For trypsin treatment and microsome enrichment, 50 μg of total protein was incubated with 10 U of DNaseI, RNase free (Fermentas) in a final volume of 100 μL of buffer A for 30 min at 30 °C. Then 5 µg of trypsin (T8003; Sigma-Aldrich) was added, and protein digestion was conducted for 30 min at room temperature. The volume was then adjusted to 1 mL with cold buffer A containing protease inhibitors (1 mM 4-(2-aminoethyl)benzenesulfonyl fluoride hydrochloride and 1 mM benzamidine), and samples were centrifuged at 100,000 × *g* for 1 h at 4 °C to generate a microsome-enriched fraction. Microsomes were resuspended in 30 µL of buffer A, and one-half of the sample was loaded on an analytical gel.

For control treatment on membrane-disrupted microsomes, 50 μg of total protein fraction was adjusted to a volume of 100 μL with buffer A and incubated with 10 U of DNaseI for 30 min at 30 °C, followed by centrifugation at 12,500 × *g* for 20 min at 4 °C. The membrane pellet was resuspended in 30 μL of buffer A supplemented with 0.2% Triton-X100, with or without trypsin (0.1 μg trypsin/μg protein) and then incubated at room temperature for 30 min.

### Endoglycosidase H Treatment.

293TN cells transfected with pSelExpress-SEPN1^U428C^ were lysed in the buffer containing 150 mM NaCl, 20 mM HEPES pH 7.5, 10 mM EDTA and 1% Triton X100, and supplemented with protease inhibitors cocktail (Roche). Buffer exchange was performed on PD-10 column (GE Healthcare) using a gravity protocol as described by the manufacturer, and samples were eluted with PBS. A portion of the eluate (36 μL) containing 50 μg of total protein was supplemented with 4 μL of 10× glycoprotein denaturating buffer (New England BioLabs) and heated at 100 °C for 10 min. One-half of the resulting sample was then treated with recombinant endoglycosidase H (EndoH; New England BioLabs) with 1,000 U of enzyme in 30 μL of total reaction volume, supplemented with 10× GlycoBuffer 3 (New England BioLabs), for 1 h at 37 °C. A parallel control reaction was performed under the same conditions but without EndoH. The samples were then mixed with 4× Laemmli buffer and analyzed by Western blotting. Biological duplicates were performed for this assay.

### Bioinformatic Analysis.

SEPN1 topology was analyzed by several secondary structure prediction algorithms, including SOSUI (version 1.11; harrier.nagahama-i-bio.ac.jp/sosui/sosui_submit.html), SPLIT (version 4.0; splitbioinf.pmfst.hr/split/4/), PSIPRED (version 4.0; bioinf.cs.ucl.ac.uk/psipred/), and TMHMM (version 2.0; www.cbs.dtu.dk/services/TMHMM/). A FASTA sequence corresponding to human SEPN1 isoform 2 from the UniProt database (https://www.uniprot.org/uniprot/Q9NZV5-2.fasta) was used as an input.

### Peptide Synthesis.

Peptides corresponding to the SEPN1 sequence (residues from T67 to S102) were synthesized by solid-phase chemistry using fluorenylmethyloxycarbonyl group-protected amino acid with Syro-I peptide synthesizer (Biotage) at 0.1 mM scale. Solutions containing the peptides purified by reverse-phase high-pressure liquid chromatography (>95% purity) were freeze-dried, and the powder was stored at −80 °C until use.

### Circular Dichroism (CD).

Lyophilized synthetic peptides were dissolved in calcium-free buffer (Tris/acetate 2 mM, pH 7.5) and adjusted to 33 μM concentration. Far-UV CD spectra were recorded on a Jasco J-815 CD spectropolarimeter at 25 °C at wavelengths of 190 to 260 nm, in a quartz cuvette with a 1-mm optical pathway (Hellma 110-QS). The following conditions were used: resolution, 0.1 nm; bandwidth, 1.0 nm; sensitivity, 100 mdeg; response, 16 s; speed, 20 nm/min; and accumulation, 1.

All CD measurements were made in triplicate using three different batches of synthetic peptides. Buffer signal was subtracted, and the resulting spectra were normalized at 260 nm. Calcium titration experiments were performed with increasing calcium concentrations (0, 0.01, 0.03, 0.1, 0.3, 1, 3, and 5 mM). The dissociation constant of the EF-hand peptide complex with calcium (*K*_d_[Ca^2+^]) was calculated using the Hill equation (with the Hill constant equal to 1). The equation was solved for CD values at 222 nm by estimating initial values of molar ellipticity, [θ]max and [θ]min, and finding the best fits using a nonlinear least squares curve-fitting method. To deconvolute the secondary structural types present in the peptide spectra, CD data were analyzed using the DichroWeb server (dichroweb.cryst.bbk.ac.uk/html/process.shtml) with the K2D algorithm (32).

### Protein Digestion and Mass Spectrometry.

Proteins were FLAG-immunoprecipitated after transfection of plasmids in SEPN1 KO HeLa cells previously treated with Tg or DMSO. The proteins were resolved on a nonreducing 10% SDS/PAGE gel and stained with Coomassie blue. The whole lane was divided into three slices that were excised, reduced by 10 mM DTT, alkylated by 55 mM NEM, and digested overnight by trypsin. Acidified peptide mixtures were desalted and concentrated on StageTipC18 (33) and injected as technical replicates on a nLC-ESI-MS/MS quadrupole Orbitrap QExactive-HF mass spectrometer (Thermo Fisher Scientific). Two injections per sample were performed as technical replicates.

For the analysis of cysteine modifications in SEPN1^U428C^, bands corresponding to monomer and oligomer of the protein were excised and first treated with 55 mM NEM to alkylate the reduced cysteines. Then disulfide bonds were reduced by 10 mM DTT and alkylated by 55 mM IAA. Protein was double-digested first by trypsin overnight and then by Asp-N; the acidified peptide mix was treated as described above. Proteins were identified and quantified, with raw files processed using MaxQuant. Raw files of the proteomic data together with all peptides identified and parameters used for the analysis were deposited into the PeptideAtlas repository (PASS01535).

### Confocal Microscopy and Ratiometric Image Analysis.

Confocal ratiometric microscopy was performed with a Nikon A1 confocal scanning unit with a 40× objective at 1.49 zoom, managed by NIS elements software. Images at 512 × 512 pixels were obtained using laser excitation of 405 or 488 nm, and emission light was collected with a 525/50 nm filter, with a sequential scanning mode to avoid bleed-through effects. Random fields of view with 7 to 10 cells per condition were acquired longitudinally and analyzed as follows. Image analysis was done using the Ratio Plus ImageJ plugin. In brief, we manually traced the cells to define quantification regions of interest. For each channel, namely Ex_405_Em_525_ and Ex_488_Em_525_, we normalized background noise and applied a correction factor to have an intensity ratio for Ex_405_Em_525_/Ex_488_Em_525_ of ∼1 at baseline. The same correction factor was applied to calculate the channel intensity ratio after background normalization at subsequent time points. Five biological replicates were performed for this assay.

### Statistics.

All data were analyzed using GraphPad Prism 7 software.

## Supplementary Material

Supplementary File

## Data Availability

Data supporting the findings of this study are provided in the main text and *SI Appendix*. Raw data have been deposited in the Zenodo public repository (https://doi.org/10.5281/zenodo.3874431).
