# Supplementary material for: Selenoprotein N is an endoplasmic reticulum calcium sensor that links luminal calcium levels to a redox activity
Source: Proc Natl Acad Sci U S A. 2020 Aug 17;117(35):21288–98. doi: 10.1073/pnas.2003847117 (PMC7474598; doi:10.1073/pnas.2003847117)
Supplement: Supplementary File [file pnas.2003847117.sapp.pdf]

Selenoprotein N is an endoplasmic reticulum calcium sensor that links luminal calcium levels to a redox activity

Alexander Chernorudskiy[a], Ersilia Varone[a#], Sara Francesca Colombo[b#], Stefano Fumagalli[a], Alfredo Cagnotto[a], Angela Cattaneo[c], Mickael Briens[d°], Mireille Baltzinger[d], Lauriane Kuhn[d], Angela Bachi[e], Andrea Berardi[f], Mario Salmona[a], Giovanna Musco[f], Nica Borgese[b], Alain Lescure[d*] & Ester Zito[a*]

[a]Istituto di Ricerche Farmacologiche Mario Negri IRCCS, Milan, Italy

[b]Consiglio Nazionale delle Ricerche Institute of Neuroscience and BIOMETRA Department, Università degli Studi di Milano, Milan, Italy

[c]Proteomics/MS Facility, Cogentech SRL Benefit Corporation, Milan, Italy

[d]University of Strasbourg, CNRS, Architecture and Reactivity of RNA, Strasbourg, France

[e]IFOM-FIRC Institute of Molecular Oncology, Milan, Italy

[f] Biomolecular NMR c/o IRCCS Ospedale S. Raffaele Via Olgettina 58 20132, Milan, Italy

° Present address: ADISSEO FRANCE SAS, Commentry, France

[#] EV and SC equally contribute

*** EZ and AL are senior authors**

Running title: Selenoprotein N is an ER calcium sensor

Corresponding author:

Ester Zito,
ORCID ID: 0000-0001-7786-7698
Istituto di Ricerche Farmacologiche Mario Negri IRCCS
Via Mario Negri 2,
20156 Milano, Italy
Tel: +39 0239014480
E-mail: ester.zito@marionegri.it

Supplementary Results

Results related to Supplementary Figure 1

We confirmed the glycosylation pattern of SEPN1 by heterologous expression of human (hSEPN1) and zebrafish (zSEPN1) SEPN1 in *Pichia pastoris* followed by mass-spectrometric analysis. Both zSEPN1 and hSEPN1 recombinant proteins were successfully expressed and secreted in the supernatant after induction with methanol, and purified by immobilized metal ion affinity chromatography (IMAC). The produced SEPN1 proteins appeared as a major band or as a doublet on SDS–PAGE migrating, around 72 kDa (Supp Fig. 1A), higher than the expected mass for the recombinant proteins (56 and 57kDa for zSEPN1 and hSEPN1, respectively), indicating the presence of post-translational modifications.

Bio-informatics analysis predicted the presence of two NxS/T consensus sites for N-linked glycosylation in zSEPN1 at positions Asn451 and Asn499, and four putative glycosylation sites in hSEPN1 at positions Asn156, Asn449, Asn471 and Asn497. Studies of nLC-MS/MS spectrometry allowed the peptides assignment to the entire zSEPN1 sequence except two peptides flanking the two predicted glycosylation sites. Analyses using mannose and N-acetylglucosamine (GlcNac) as diagnostic ions confirmed the glycosylation at both sites. The glycosylation of the high mannose type was characterized as two GlcNac and ten mannoses on Asn451 and two GlcNac and fourteen mannoses on Asn499 (Supp Fig. 1B). The MS/MS spectra obtained on the human protein revealed the modification of Asn156 with two GlcNac and eleven mannoses and the probable modifications of the two conserved Asn449 and Asn497 as the corresponding tryptic peptides were not detected at their expected unmodified mass. Finally, the second non-conserved site in hSEPN1 Asn471 was unambiguously identified to be not modified. Altogether, the presence of N-glycosylations on the Asn of SEPN1 is in agreement with its intraluminal localization.

A

Sup. Fig. 1

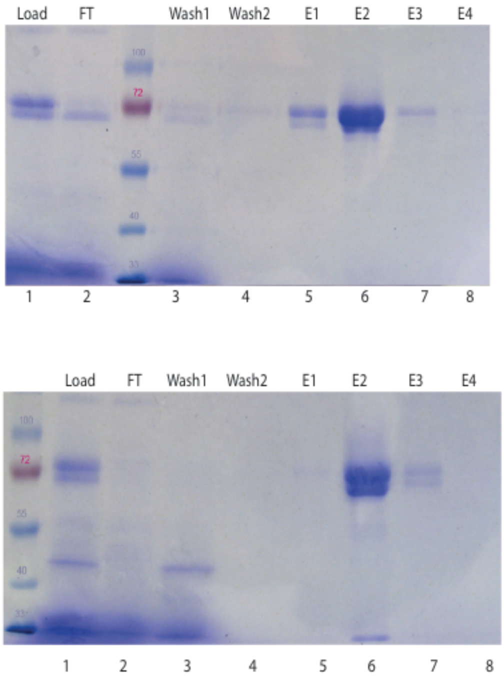

Load
FT
Wash1
Wash2
E1
E2
E3
E4
1
2
3
4
5
6
7
8
Load
FT
Wash1
Wash2
E1
E2
E3
E4
1
2
3
4
5
6
7
8

B

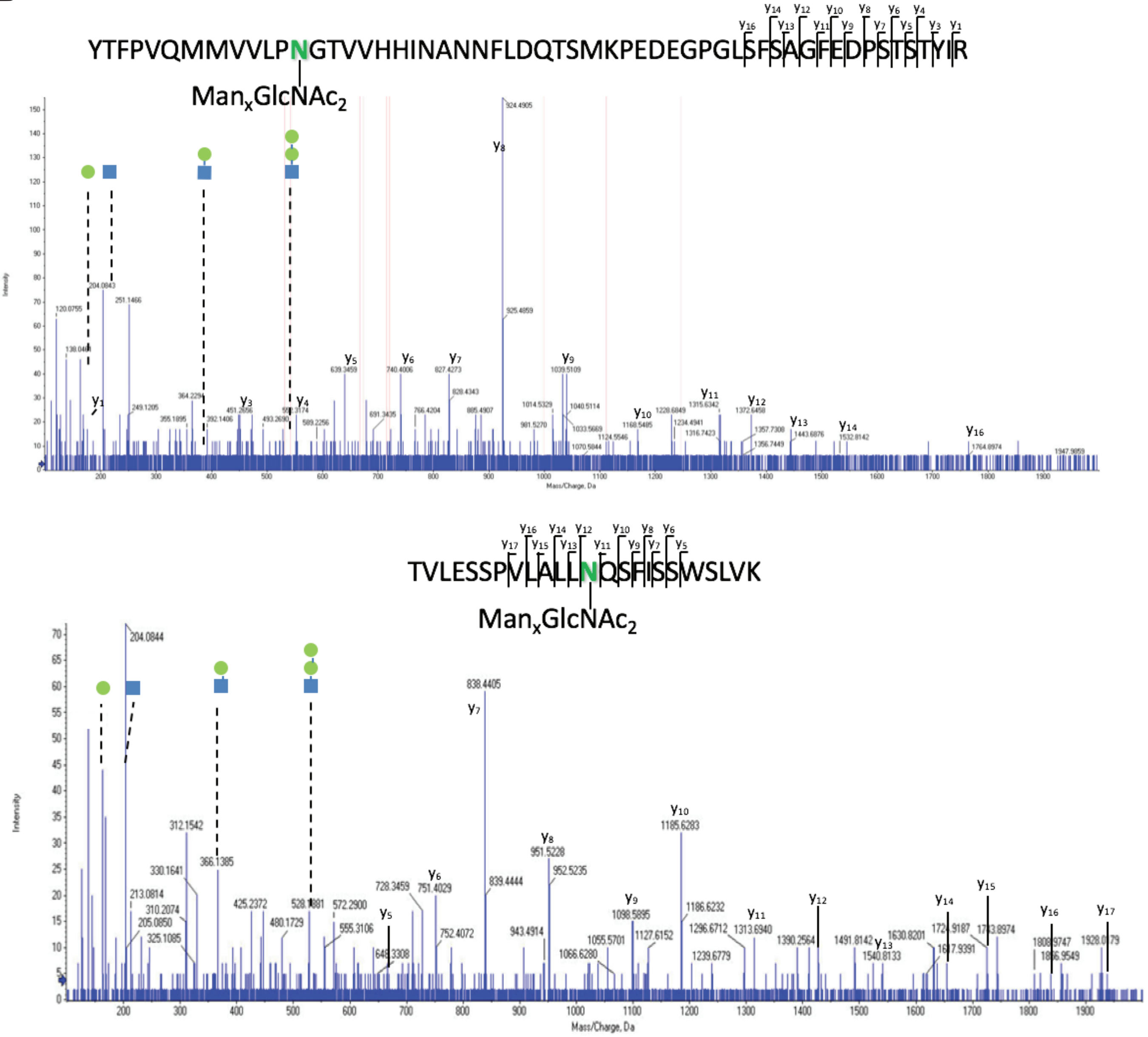

YTFPVQMMVVLPNGTVVHHINANNFLDQTSMKPEDEGPGLSFSAGFEDPSTSTYIR
Man$_x$GlcNAc$_2$
Intensity
Mass/Charge, Da
TVLESSPVLALLNQSFISSWSLVK
Man$_x$GlcNAc$_2$
Intensity
Mass/Charge, Da

Supplementary Figure 1

A) Expression of zebrafish (zSEPN1) and human (hSEPN1) proteins in the yeast *Pichia pastoris* expression system. Selenocysteine to cysteine mutants of zSEPN and hSEPN sequences were cloned into the pPICZαA vector (Invitrogen™). In these constructs, the SEPN1 N-terminal sequence, including the transmembrane domain, was substituted by the yeast α-factor signal sequence, that allowed for efficient secretion in the medium of the recombinant protein. The two constructs were transformed into yeast *Pichia pastoris* GS115. The culture media containing the secreted recombinant zSEPN1 (upper panel) and hSEPN1 (lower panel) proteins and the nickel-affinity chromatography purified fractions were analyzed by SDS-PAGE. The expressed proteins were abundantly represented in the culture media (lanes 1). Both proteins were retained on the column, as a band around 72 kDa absent in the flow-through fractions (lanes 2). After two washes, zSEPN1 or hSEPN1were eluted from the column using imidazole (lanes 5 to 8). A single band was obtained for the purified zSEPN1, while purified hSEPN1 appeared as a doublet.

B) LC-MS/MS analysis of the modified peptides from purified zSEPN1. The two peptides, which sequences are depicted on the top, correspond to tryptic peptides including residues Asn451 (upper panel) or Asn499 (lower panel) of zSEPN1. Both peptides are glycosylated according to their intact mass and the MS/MS fragmentation pattern from the precursor peptide at m/z=1111.64 (8+, eluted at 43.52min) and at m/z= 1161.80 (4+, eluted at 59.57min) respectively. We can confidently deduce that these peptides are modified by a high-mannose N-glycosylation due to (i) the sequence of 11 and 12 consecutive amino acids matching only on SEPN1 protein, (ii) the presence of a single consensus site NxS/T in each tryptic peptide, and (iii) the detection of four diagnostic glycan ions in the two patterns (immonium ions displayed on the spectra according to the international nomenclature: circle = Hex, square = HexNAc).

Sup. Fig. 2

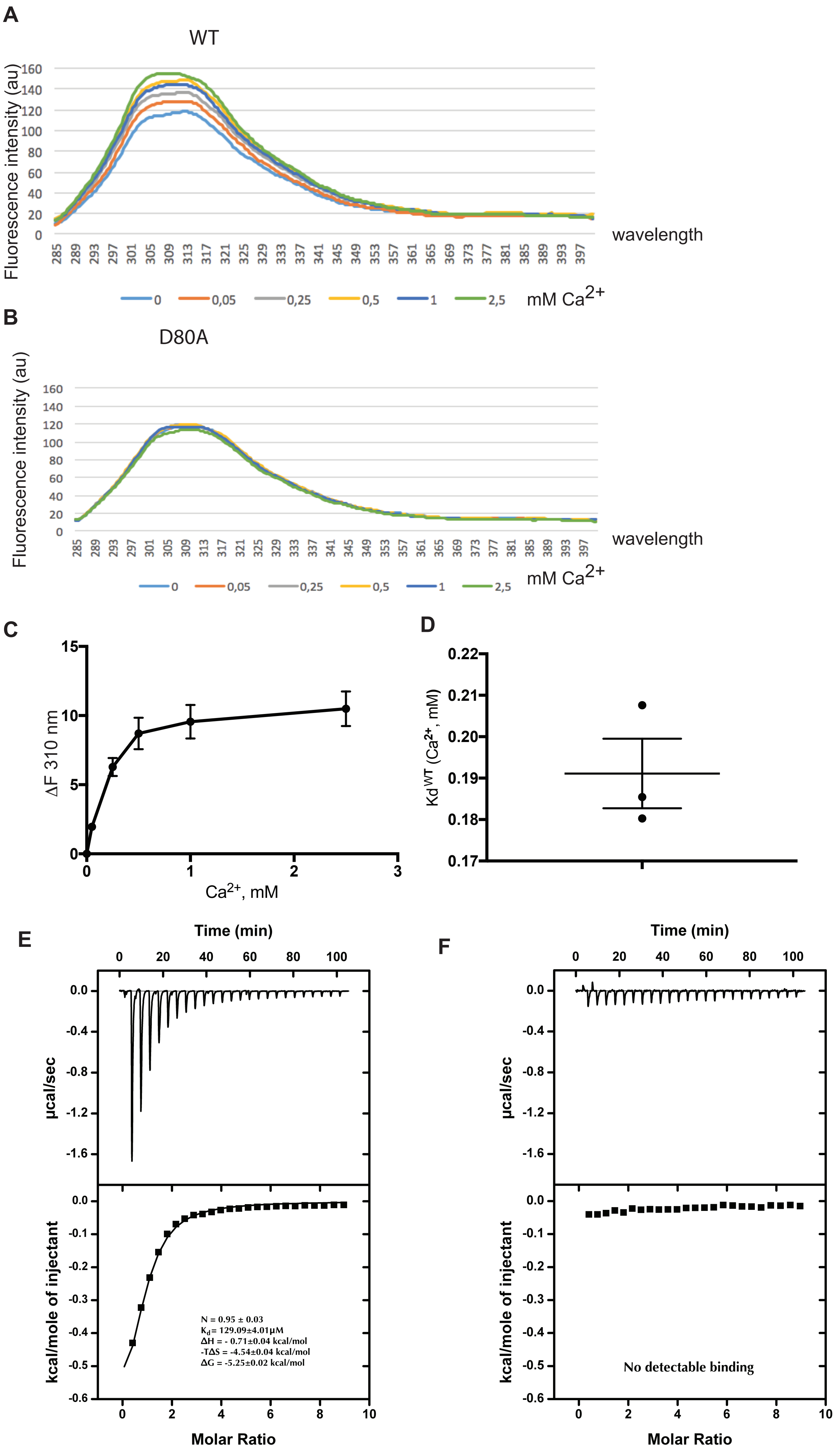

A
WT
Fluorescence intensity (au)
wavelength
mM Ca2+
0
0,05
0,25
0,5
1
2,5
B
D80A
Fluorescence intensity (au)
wavelength
mM Ca2+
C
ΔF 310 nm
Ca2+, mM
D
KdWT (Ca2+, mM)
E
Time (min)
µcal/sec
kcal/mole of injectant
Molar Ratio
N = 0.95 ± 0.03
Kd= 129.09±4.01µM
ΔH = - 0.71±0.04 kcal/mol
-TΔS = -4.54±0.04 kcal/mol
ΔG = -5.25±0.02 kcal/mol
F
Time (min)
µcal/sec
kcal/mole of injectant
Molar Ratio
No detectable binding

Supplementary Figure 2

A) Tyrosine fluorescence spectra of WT and B) D80A peptide at different calcium concentrations. C) Plot of calcium concentrations vs changes in fluorescence intensity at single wavelength (310 nm). D) Kd^{Ca2+} of WT calculated from tyrosine fluorescence values at 310 nm in three different experiments. ITC-binding curves of $CaCl_2$ to E) WT and F) D80A. The upper and lower panels show the raw and integrated data of the sequential heat pulses of a representative experiment, respectively. Integrated data were corrected for heat of dilution and fit to a single-site-binding model using a nonlinear least-squares method (line). The stoichiometry (N) and the thermodynamic parameters are the mean of the triplicates ± sem.

Sup. Fig. 3

A

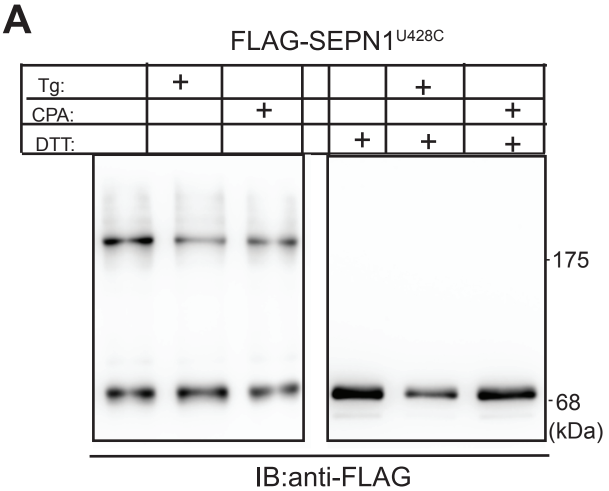

	Monomer/ oligomer
DMSO:	1
Tg:	2,08
CPA:	1,32

B

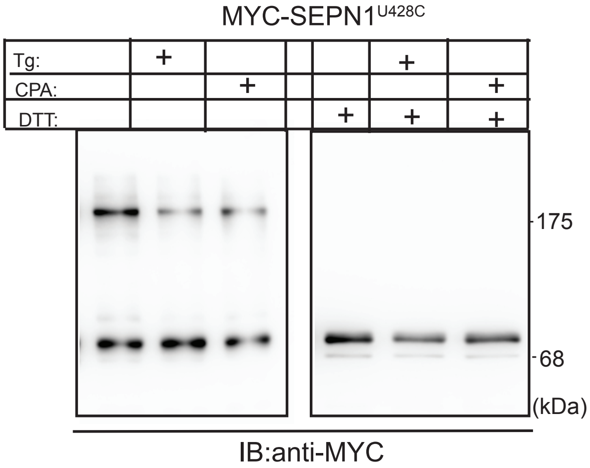

	Monomer/ oligomer
DMSO:	1
Tg:	2,98
CPA:	1,87

C

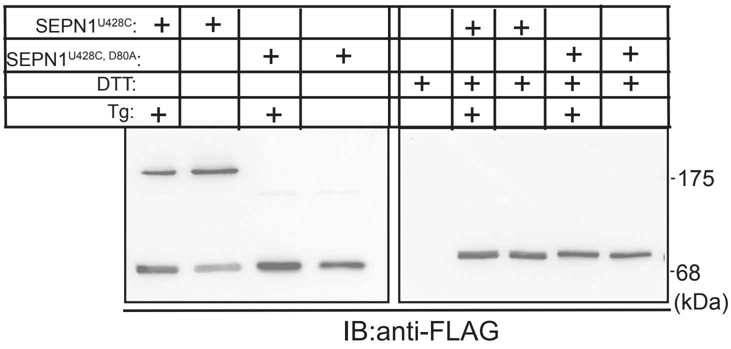

Supplementary Figure 3

A) Non-reducing and reducing immunoblot of cells transfected with FLAG-SEPN1 or with B) MYC-SEPN1 and exposed to the irreversible (thapsigargin) or the reversible (CPA) SERCA inhibitors. The two tables on the right indicate the monomer/oligomer ratio in arbitrary units.

C) Reducing and non-reducing Immunoblot of the input protein lysate of the sucrose gradients in Figure 3C.

Sup. Fig. 4

Gene ontology of biological process

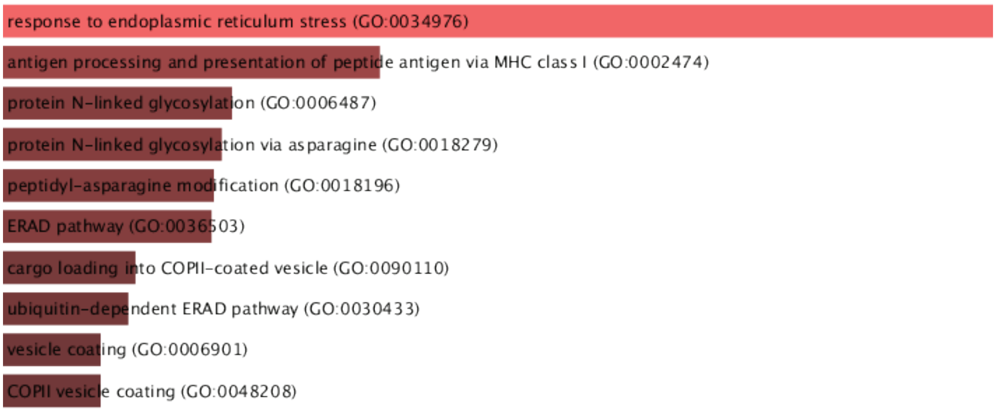

Name	Adjusted p-value
Response to endoplasmic reticulum stress (GO:0034976)	1.261e-10
Antigen processing and presentation of peptide antigen via MHC class I (GO:0002474)	0.000004392
Protein N-linked glycosylation (GO:0006487)	0.00003898
Protein N-linked glycosylation via asparagine(GO:0018279)	0.00003898
Peptidyl-asparagine modification (GO:0018196)	0.00003586
ERAD pathway (GO:0036503)	0.00003132
Cargo loading into COPII-coated vesicle (GO:0090110)	0.0001068
Ubiquitin-dependent ERAD pathway (GO:0030433)	0.0001066
Vesicle coating (GO:0006901)	0.0001563
COPII vesicle coating (GO:0048208)	0.0001407

Supplementary Figure 4

Pathway annotation of proteins interacting with SEPN1$^{C427S, U428C}$ after ER calcium depletion (Tg) by Gene Ontology (GO) program searching for biological process and below, related table with the adjusted p-value. Proteins belonging to the ER stress response pathway stand out as indicated by the lowest p-value in the table.

A

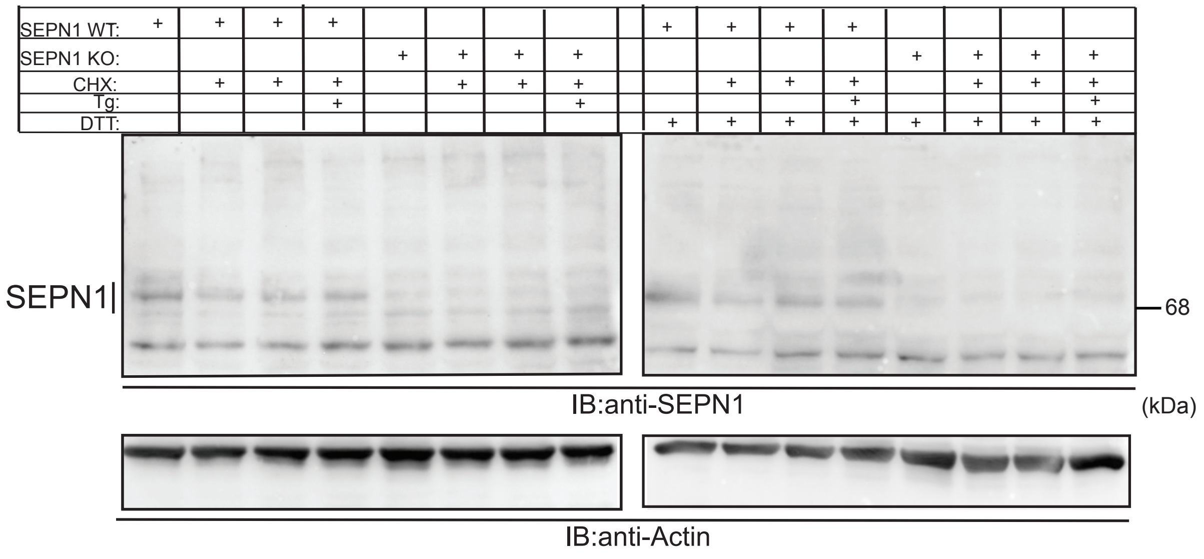
SEPN1 WT:
SEPN1 KO:
CHX:
Tg:
DTT:
SEPN1
68
IB:anti-SEPN1
(kDa)
IB:anti-Actin

B

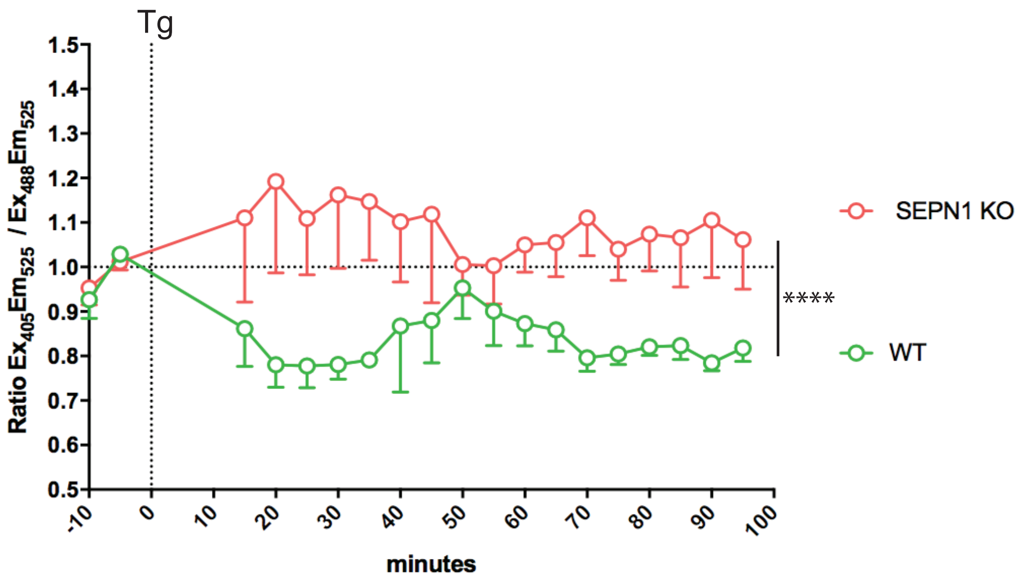
Tg
Ratio Ex405Em525 / Ex488Em525
1.5
1.4
1.3
1.2
1.1
1.0
0.9
0.8
0.7
0.6
0.5
-10
0
10
20
30
40
50
60
70
80
90
100
minutes
****
SEPN1 KO
WT

Supplementary Figure 5

A) Non-reducing and reducing immunoblot of endogenous SEPN1 in lysates of WT and SEPN1 KO HeLa cells with the indicated treatments. An anti Actin blot serves as the loading control.

B) Traces of time-dependent changes in the fluorescence excitation ratio of roGFP2, reflecting the alterations in the redox state of roGFP2 localized in the ER of WT and SEPN1 KO cells exposed to Tg. The trace represents data from 5 different experiments (N=50 cells, two-way ANOVA, P<0,001).

SUPPLEMENTARY METHODS

Expression constructs

Expression plasmids encoding FLAG-tagged human SEPN1 (WT protein and active site mutants C427S/U428C and C427S/U428S) in pSelExpress vector were previously described (1). Cells transfected with pSelExpress-SEPN1 WT were cultured in the presence of 500 nM sodium selenite to facilitate the expression of full-length SEPN1. Same WT sequence was also subcloned into pcDNA3.1 vector (Invitrogene) and further used for mutagenesis. Single amino acid substitutions in SEPN1 sequence (C49S, D80A, M85V, Y86C, C108S, C243S, U428C) in either pcDNA3.1 or pSelExpress expression vectors were made using Agilent QuikChange II XL Site-Directed Mutagenesis Kit according to manufacturer's instructions.

For creation of His-tagged SEPN1 construct, the sequence coding for a U428C mutant of human protein was PCR amplified and cloned into the pQE-TriSystem vector (Qiagen), resulting in SEPN1 construct with 8xHis-tag at C-terminus. In parallel, primers that introduced a StrepII-tag at the N-terminus were used to amplify the same sequence, and the PCR fragment was cloned into the eukaryotic expression vector pXJ41 (2).

The pcDNA3 roGFP2 plasmid encoding SS_FLAG_roGFP2 (where SS is an artificial signal sequence) under control of CMV promoter was a gift from David Ron.

Mutagenesis

Single amino acid substitutions in SEPN1 sequence (C49S, D80A, M85V, Y86C, C108S, C243S, U428C) in either pcDNA3.1 or pSelExpress expression vectors were made using Agilent QuikChange II XL Site-Directed Mutagenesis Kit according to manufacturer's instructions. The mutagenic oligonucleotide primer pairs for each substitution are given in a table below (all sequences in 5'-3' order). All resulting constructs were verified by Sanger sequencing.

**Mutant**	**Forward primer**	**Reverse primer**
C49S	Tgcctagcggagactctcacggctgca	Tgcagccgtgagagtctccgctaggca

D80A	Catgtctccatcggtggccagggagctaaacag	Ctgtttagctccctggccaccgatggagacatg
M85V	Tcaggagagatgtacacgtctccatcggtgtcc	Ggacaccgatggagacgtgtacatctctcctga
Y86C	Attcctcaggagagatgcacatgtctccatcggtg	Caccgatggagacatgtgcatctctcctgaggaat
C108S	Ttcctcttcctcagaagatgcagcaggtgtgg	Ccacacctgctgcatcttctgaggaagaggaa
C243S	Tagcggtcagggatgcgacagcgcc	Ggcgctgtcgcatccctgaccgcta
U428C	Agttcgtccggatccgcagcatgactgatcg	Cgatcagtcatgctgcggatccggacgaact

Cell culture and transfection

SEPN1 KO HeLa cells were generated by using CRISPR/Cas9 technology (Origene) following manufacturer's guidelines. pCas-Guide constructs encoding Cas9 and custom guide RNA sequence (GAACTGGCGCTGAAGACCCT) targeting exon 2 of human SEPN1 gene were ordered from Origene. Subconfluent HeLa cells were transfected in 6cm Petri dishes with 5 ug of pCas-Guide DNA using OptiMEM medium (Gibco) and FuGENE HD transfection reagent (Promega) at 1:3 DNA:reagent ratio according to manufacturer's manual. Transfection medium was substituted with normal growth medium 16 hours post transfection. 72 hours post transfection cells were collected, lysed and analyzed by Western blotting for Cas9 expression. Expression of SEPN1 was analyzed by RT-PCR. Individual clones were isolated by diluting the transfected cellular pool and seeding in 96-well plate at a density of <1 cell per well. Clones were grown for 1 week before further analysis. Genomic DNA was isolated from clones in 6-well plates. PCR using genomic DNA as a template was performed with oligonucleotide primers specific for introns surrounding exon 2 of SEPN1 gene (forward: 5'-ctcaggaagatggtgggaga-3'; reverse: 5'-ctcagtgaagacaccgttgg-3'). Introduction of InDels destroying the correct SEPN1 sequence was verified by sequencing of purified PCR products.

293TN and HeLa cells were cultured in Dulbecco's modified Eagle's medium (DMEM, Gibco) supplemented with 2 mM glutamine, 10% fetal bovine serum and 1% penicillin/streptomycin in a humidified atmosphere of 5% CO_2 at 37°C.

The cells were transfected at 20-50% confluence with FuGENE HD transfection reagent (Promega) using optimized conditions. Briefly, 3 ul of reagent were used for every 1 ug of DNA, and the transfection mix was prepared in Opti-MEM (Gibco). 16 hours post transfection the medium was replaced with complete DMEM.

For experiment with His- and Strep-tagged SEPN1, cells were grown in 6-well plates and transiently transfected at 80% confluency with Nanofectine (PAA, GE Healthcare) using modified manufacturer's recommendations. Notably, 3 µl of Nanofectine were used for 2 µg total vector DNA per well of a 6-well plate. Twenty-four hours post-transfection cells were washed with phosphate buffered saline (PBS) and cultured as described above.

Western blotting

Cells were lysed in cold buffer containing 150 mM NaCl, 20 mM HEPES pH 7.5, 10 mM EDTA and 1% Triton X100, and supplemented with protease inhibitors cocktail (Roche) and 20 mM NEM. Protein concentration was determined by standard BCA assay (Pierce). Samples with equal protein concentration were mixed with non-reducing Laemmli buffer (62.5 mM Tris-HCl pH 6.8, 2% SDS, 10% glycerol and 0.01% bromophenol blue) and heated for 5 min at 95°C. For reducing SDS-PAGE, samples were supplemented with 100 mM DTT. Protein samples separated by either reducing or non-reducing SDS-PAGE were then transferred to Protran nitrocellulose membrane (Merck) and probed with the following antibodies: mouse pan-actin from Sigma Aldrich (clone C4, 1:5000), Selenoprotein N (A-11, 1:1000) and SERCA2 (F-1, 1:1000) from Santa Cruz Biotechnology, anti-His (1:4000) from Pierce, or mouse anti-Strep (1:2000) from IBA BioTAGnology.

Immunoprecipitation

Cells were lysed in cold lysis buffer supplemented with protease inhibitors cocktail (Roche) and 20 mM NEM. Samples containing 1-2 mg of total protein were pre-cleared using SureBeads protein G magnetic beads (Bio-Rad Laboratories) for 1 hr and incubated with 20-30 ul of EZview Red anti-FLAG M2 affinity gel (Sigma-Aldrich) for 16 hrs at 4°C. Beads were than washed 4 times with lysis buffer, and immunoprecipitated proteins were detached from beads by heating to 70°C for 5 min in

2x non-reducing Laemmli buffer. All immunoprecipitation-based assays were performed in biological triplicate.

Sucrose gradient

Subconfluent HeLa cells were transfected and after 40 hrs were treated with cycloheximide (CHX, Alfa Aesar) at 50 ug/ml in complete growth medium for 6 hrs. CHX was used as an inhibitor of protein synthesis in order to decrease the amount of monomeric/oligomeric SEPN1 species preexisting to the following treatment. After CHX washout, cells were incubated with 1 uM thapsigargin (Tg) or DMSO as mock for 2 hours under standard growth conditions, then gently washed in PBS and collected by scraping in cold PBS. Cells were pelleted at 1200 g for 3 min (4°C) and subsequently incubated with PBS, NEM 20 mM (5 min on ice) and then washed twice with cold PBS and once with PBS supplemented with protease inhibitors cocktail. Pelleted cells were lysed in a buffer containing 20 mM NaCl, 25 mM TrisHCl pH 7.4 and 1% Triton X100 and supplemented with protease inhibitors and subsequently the unsoluble material pelleted at 16000 g for 10 minutes. Supernatant was collected after centrifugation and loaded on top of linear sucrose gradient (5-20% sucrose in a buffer containing 20 mM NaCl, 25 mM TrisHCl pH 7.4 and 0.2% Triton X100) prepared in 2-chamber gradient mixer. Centrifugation was performed in Beckman Optima 90 centrifuge equipped with SW-41 rotor, at 37,000 rpm for 17 hrs (4°C). After centrifugation, 19 individual fractions for each sample were collected by peristaltic pump. Each fraction was precipitated by TCA and redissolved in a small volume of PBS. Samples were then mixed with Laemmli buffer and loaded on SDS-PAGE. Fluorescent Western blotting was used to visualize FLAG-tagged proteins, with anti-FLAG M2 (1:1000) as primary antibody and IRDye 680RD goat anti-Mouse IgG (Li-Cor, 1:15000) as secondary antibody. Fluorescent signal from dried membranes was acquired on Li-Cor Odyssey instrument and quantified by Li-Cor Image Studio software. Sucrose gradients were performed in biological duplicates.

Immunofluorescence

Cells grown in ibidi µ-Slide 8 well were fixed in a complete growth medium supplemented with 3.7% formaldehyde for 15 min at 37°C. After several washes with PBS, cells were permeabilized by 0.2% Triton X100 in PBS, 10 min at room temperature (RT). Blocking of non-specific binding was performed by incubation with 20% normal goat serum (Vector Laboratories), 30 min RT. Primary antibody (mouse anti-PDI, clone 1D3, Enzo) was diluted at 1:200 in blocking solution. Secondary antibody (goat anti-Mouse IgG Alexa Fluor 546, ThermoFisher) were used at 1:500 dilution. Samples were incubated sequentially with primary and secondary antibodies for 1 hr at RT, with several washes with PBS / PBS + 0.5% Tween 20. Nuclei were stained with Hoechst 33342 (ThermoFisher) diluted to 1 µg/ml in PBS, 5 min RT. Images were acquired by confocal microscopy (Nikon A1) as previously described (3). Immunofluorescence assay was performed in two experimental replicates.

Peptide synthesis

Peptides corresponding to SEPN1 sequence (residues from T67 to S102) were synthesized by solid-phase chemistry using FMOC (fluorenylmethyloxycarbonyl) group protected amino acid with Syro-I peptide synthesizer (Biotage) at 0.1 mM scale. Merck Nova Syn TGA resin (0.26 mmol/g) was used. The FMOC group was automatically removed using 22% of piperidine in N-methylpyrrolidone solution and amino acids activated with TBTU (O-(benzotriazol-1-yl)-N,N,N,N-tetramethyluronium-tetrafluoroborate) and N,N,-diisopropylethylamine. After the last coupling cycle of each amino acid, a capping step with acetic anhydride was performed. Peptides were then cleaved from the resin with a cocktail of TFA (trifluoroacetic acid) and TIS (triisopropylsilane) (95:5 vol/vol) for 3 hours, precipitated and washed with cold diethyl ether 3 times. Subsequently the peptides were purified by reverse phase HPLC on a semi-preparative C18 column with mobile phases of 0.1% TFA in water (eluent A) and 0,08% TFA in acetonitrile (eluent B), using a linear gradient from 5 up to 100% of eluent B in 60 min. The peaks were collected and accurate masses of peptides were recorded by MALDI-TOF mass spectrometer (ABI 4800, Applied Biosystems) operating in reflector mode. The solutions containing the peptides with a purity greater than 95% were finally freeze-dried and the powder stored at -80°C until use.

Intrinsic tyrosine fluorescence

Lyophilized synthetic peptides were dissolved in calcium-free buffer (Tris/acetate 2 mM, pH 7.5) and adjusted to 33 uM concentration. Fluorescent spectra were recorded on Perkin Elmer LS-50B spectrophotometer with FL WinLab software, at room temperature in a quartz cuvette with 10 x 4 mm optical pathway (Hellma 108-QS). The following conditions were used: emission wavelengths range, 285 – 400 nm; excitation wavelength, 274 nm; and scan speed, 60 nm/min.

All measurements were made at least in triplicate using two different batches of synthetic peptides. Buffer signal was subtracted from the resulting spectra. Calcium titration experiments were performed with increasing calcium concentrations (0; 0.05; 0.25; 0.5; 1; 2.5 mM). The dissociation constant of the EF-hand peptide complex with calcium (Kd[Ca^{2+}]) was calculated by using Hill equation (Hill constant equal to 1). The equation was solved for fluorescence intensity values at 310 nm by finding the best fits using non-linear least squares curve fitting method.

Isothermal titration calorimetry

ITC titrations were performed at 23°C on a VP-ITC isothermal titration calorimeter (MicroCal LLC, Northampton, MA, USA). Synthetic SEPN1 peptides (WT and D80A) and $CaCl_2$ were dissolved in the same buffer (2mM Tris pH 7.5). To measure the Kd between SEPN1 peptides and $CaCl_2$ stepwise injections of 3.5 mM $CaCl_2$ solution (up to a a 9-fold molar excess of $CaCl_2$, with respect to SEPN1 peptides) into a cell containing a 0.3 mM SEPN1 peptides were performed. The quantity of heat absorbed or released in the process was measured. Control experiments were performed under identical conditions to determine the dilution heat of the titrant $CaCl_2$ into buffer and of the buffer into buffer. The experiments were repeated 3 times and the data were analysed with the software ORIGIN 7.0.

Mass spectrometry analysis

Peptides were separated on a linear gradient from 95% solvent A (2% ACN, 0.1% formic acid) to 50% solvent B (80% acetonitrile, 0.1% formic acid) over 36 min and from 50 to 100% solvent B in 2 min at a flow rate of 0.25 µl/min on UHPLC Easy-nLC 1000 (Thermo Scientific) connected to a 25-cm fused-silica emitter of 75 µm inner diameter (New Objective, Inc. Woburn, MA, USA), packed in-house with ReproSil-Pur C18-AQ 1.9 µm beads (Dr Maisch Gmbh, Ammerbuch, Germany) using a high-pressure bomb loader (Proxeon, Odense, Denmark).

Peptides deriving from the monomeric and oligomeric form of SEPN1^{U428C} were separated on a linear gradient from 95% solvent A (2% ACN, 0.1% formic acid) to 50% solvent B (80% acetonitrile, 0.1% formic acid) over 23 min and from 50 to 100% solvent B in 2 min at a flow rate of 0.25 µl/min MS data were acquired using a data-dependent top 15 method for HCD fragmentation. Survey full scan MS spectra (300–1650 Th) were acquired in the Orbitrap with 60000 resolution, AGC target 3^{e6}, IT 20 ms. For HCD spectra, resolution was set to 15000 at *m*/*z* 200, AGC target 1^{e5}, IT 80 ms; NCE 28%, isolation width 1.2 *m*/*z* and a dynamic exclusion of 20 sec.

To improve the detection of SEPN1 ^{U428C} peptides containing Cys +NEM and +IAA was applied an inclusion mass list with m/z of 2+ and 3+ for tryptic and Asp-N predicted peptides.

Proteins were identified and quantified processing raw files with MaxQuant ver. 1.5.2.8 searching against the database uniprot_cp_human_2015_03, trypsin specificity (or trypsin and AspN for SEPN1^{U428C}) and up to two missed cleavages; N-ethylmaleimide and N-ethylmaleimide + water of cysteine, oxidation of methionine were set as variable modifications. Mass deviation for MS/MS peaks was set at 20 ppm. The peptides and protein false discovery rates (FDR) were set to 0.01; the minimal length required for a peptide was six amino acids; a minimum of two peptides and at least one unique peptide were required for high-confidence protein identification.

Reverse and know contaminants hits were eliminated; LFQ Intensities of identified proteins were normalized by the corresponding SEPN1 intensity and z-score was calculated with Perseus ver. 1.5.0.31. For analysis of the modification on cysteines of SEPN1^{U428C}, raw files were processed with MaxQuant ver. 1.5.2.8 searching against the database uniprot_cp_human + SEPN1^{U428C} sequence.

References:

1. Marino M*, et al.* (2015) SEPN1, an endoplasmic reticulum-localized selenoprotein linked to skeletal muscle pathology, counteracts hyper-oxidation by means of redox-regulating SERCA2 pump activity. *Hum Mol Genet* (24):1843-1855.
2. Lescure A, Gautheret D, Carbon P, & Krol A (1999) Novel selenoproteins identified in silico and in vivo by using a conserved RNA structural motif. *J Biol Chem* 274(53):38147-38154.
3. Varone E*, et al.* (2019) SELENON (SEPN1) protects skeletal muscle from saturated fatty acid-induced ER stress and insulin resistance. *Redox Biol* 24:101176.
